# A ZIP6-ZIP10 heteromer controls NCAM1 phosphorylation and integration into focal adhesion complexes during epithelial-to-mesenchymal transition

**DOI:** 10.1038/srep40313

**Published:** 2017-01-18

**Authors:** Dylan Brethour, Mohadeseh Mehrabian, Declan Williams, Xinzhu Wang, Farinaz Ghodrati, Sepehr Ehsani, Elizabeth A. Rubie, James R. Woodgett, Jean Sevalle, Zhengrui Xi, Ekaterina Rogaeva, Gerold Schmitt-Ulms

**Affiliations:** 1 Tanz Centre for Research in Neurodegenerative Diseases, University of Toronto, Ontario, Canada; 2 Department of Laboratory Medicine & Pathobiology, University of Toronto, Ontario, Canada; 3 Whitehead Institute for Biomedical Research, Cambridge, MA, USA; 4 Lunenfeld-Tanenbaum Research Institute, Mount Sinai Hospital, Toronto, Ontario, Canada; 5 Department of Neurology, University of Toronto, Ontario, Canada

## Abstract

The prion protein (PrP) evolved from the subbranch of ZIP metal ion transporters comprising ZIPs 5, 6 and 10, raising the prospect that the study of these ZIPs may reveal insights relevant for understanding the function of PrP. Building on data which suggested PrP and ZIP6 are critical during epithelial-to-mesenchymal transition (EMT), we investigated ZIP6 in an EMT paradigm using ZIP6 knockout cells, mass spectrometry and bioinformatic methods. Reminiscent of PrP, ZIP6 levels are five-fold upregulated during EMT and the protein forms a complex with NCAM1. ZIP6 also interacts with ZIP10 and the two ZIP transporters exhibit interdependency during their expression. ZIP6 contributes to the integration of NCAM1 in focal adhesion complexes but, unlike cells lacking PrP, ZIP6 deficiency does not abolish polysialylation of NCAM1. Instead, ZIP6 mediates phosphorylation of NCAM1 on a cluster of cytosolic acceptor sites. Substrate consensus motif features and *in vitro* phosphorylation data point toward GSK3 as the kinase responsible, and interface mapping experiments identified histidine-rich cytoplasmic loops within the ZIP6/ZIP10 heteromer as a novel scaffold for GSK3 binding. Our data suggests that PrP and ZIP6 inherited the ability to interact with NCAM1 from their common ZIP ancestors but have since diverged to control distinct posttranslational modifications of NCAM1.

ZIP (Zrt-, Irt-like protein) metal ion transporters are an ancient family of multi-spanning transmembrane proteins tasked with the import of divalent cations into the cytosol^1^. Humans and mice express fourteen ZIP proteins that are coded by members of the solute carrier family 39a gene family (*Slc39a1* to *Slc39a14*)^2^. Beyond their role in divalent cation import, relatively little is known about their function, regulation or protein interactions. However, two ZIP paralogs, namely ZIP6 and ZIP10, were observed to co-enrich with the prion protein (PrP)^3^, a protein best known for its central role in neurodegenerative diseases of the same name^4^. Subsequent multidisciplinary analyses revealed that ZIP transporters and PrP not only bind to each other but share a common ancestry^5^,^6^,^7^. More specifically, the PrP founder gene can be traced to an evolutionary event that occurred during vertebrate speciation. Mechanistically, it can be deduced to have involved the retro-insertion of a spliced and C-terminally truncated transcript of an ancestral ZIP transporter with similarity to ZIP6 and ZIP10^8^. Intriguingly, these discoveries were preceded by independent observations, which indicated that both morpholino-based knockdown (kd) of ZIP6^9^ or PrP^10^ cause a similar gastrulation arrest phenotype in zebrafish embryos. A closer characterization of this shared phenotype indicated it as being caused by a failure to fully execute a morphogenetic program known as epithelial-to-mesenchymal transition (EMT)^9^,^11^. One plausible interpretation of this finding was that ZIP6 and PrP are not only homologous but PrP may have inherited from its ZIP ancestor at least part of its function. This led us to consider whether an involvement of the prion protein in EMT is conserved in mammalian cells. Consistent with this scenario, our previous work established that transcript and protein levels of the prion protein are profoundly upregulated during EMT in mouse NMuMG epithelial cells, a well-known model for studying transforming growth factor beta 1 (TGFB1)-induced EMT^12^. Moreover, the CRISPR/Cas9-based knockout (ko) of PrP partially impaired EMT in this model^13^. Comparative global proteome analyses of wild-type (wt) and PrP-deficient cells then shortlisted the neural cell adhesion molecule 1 (NCAM1), a protein previously observed to bind to PrP^14^, as a key player in EMT, whose expression is affected in PrP-deficient cells^13^. Closer analyses of NCAM1 revealed that PrP not only influences steady-state levels of NCAM1 but also controls its polysialylation. Surprisingly, this effect of PrP on a key NCAM1 modification was not reliant on PrP residing in immediate proximity to NCAM1; based on the previously documented interaction of PrP with NCAM1 (ref. 14), we expected that PrP might be essential to direct the *St8sia2* polysialyltransferase responsible for NCAM1 polysialylation to its NCAM1 substrate, while the latter is resident in the Golgi. Instead, it placed PrP upstream of a signaling loop that controls *St8sia2* transcription. Taken together, this body of work raised several interesting questions: (1) Did PrP inherit its intimate involvement in a biology that modulates the expression of NCAM1 and controls its polysialylation from its ZIP6-like ancestor? and, if so, (2) are ZIP6 and PrP equivalent in this regard, or have they acquired specialized roles with respect to their influence on NCAM1?

To address these questions, we generated ZIP6 knockout clones, investigated the functional involvement of ZIP6 in EMT, and conducted an unbiased ZIP6 interactome analysis. We show that ZIP6 transcript and protein levels are dramatically upregulated upon induction of EMT. Moreover, we observed that ZIP6 forms a heteromeric complex with ZIP10, whose predominant interactor is NCAM1. Subsequent in-depth analyses of NCAM1 interactors revealed a critical role for ZIP6 in the assembly of NCAM1-bound focal adhesion complexes. We document a minor influence of ZIP6 on the levels of polysialylated NCAM1 (PSA-NCAM1) and instead uncovered a signaling module that has ZIP6/10 collaborate closely with GSK3B in its influence on NCAM1. The data are discussed in an evolutionary context, highlighting how different selective pressures and membrane topologies may have led to specialized adaptations of ZIP6 and PrP within the same overall developmental program centered on NCAM1.

## Results

### Generation of a mammalian ZIP6 knockout model

**Figure 1:**
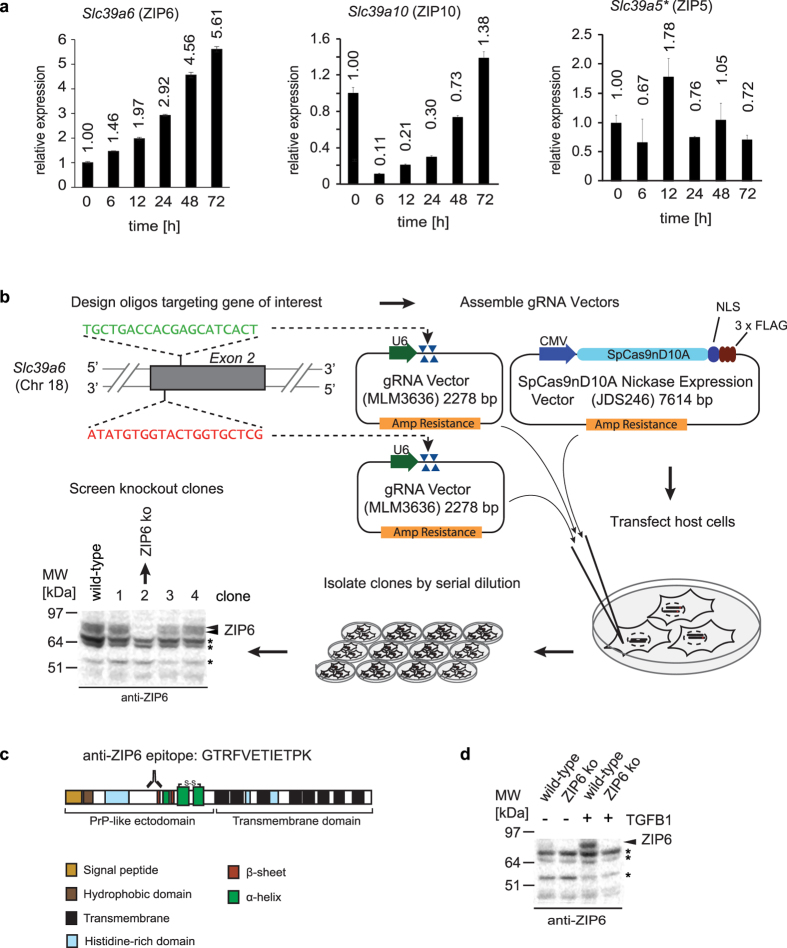
Mouse NMuMG cell model and tools for investigating role of ZIP6 during EMT. (**a**) ZIP6 transcript levels are more than five-fold increased during epithelial-to-mesenchymal transition induced in NMuMG mouse epithelial cells upon 72 h exposure to TGFB1. The asterisk in this panel designates the fact that this quantitation had to be based on relatively low levels of ZIP5 mRNAs in NMuMG cells, making this quantitation less robust than those depicted for ZIP6 and ZIP10. (**b**) Strategy employed for CRISPR-Cas9-based knockout of ZIP6 protein expression in NMuMG cells following Cas9-D10A nickase-mediated single-strand cleavages of *Slc39a6* Exon 2 at non-overlapping target sites. (**c**) Schematic of ZIP6, indicating peptide epitope of in-house generated polyclonal antibody used in this work. (**d**) Evidence of ZIP6 protein level increase in NMuMG cells upon 48 h exposure to TGFB1. Bands labeled with asterisks indicate non-specific bands.

To investigate if mouse NMuMG cells represent a suitable model for studying the involvement of ZIP6 on EMT, we initially induced this morphogenetic program in wild-type NMuMG cells by the addition of TGFB1 and measured transcript levels of ZIP6 and its closest paralogs ZIP10 and ZIP5 (Fig. 1a)^7^. Reminiscent of the previously reported transcript and protein profile of PrP in the same cell model^12^,^13^, ZIP6 transcript levels increased more than five-fold upon exposure of cells to TGFB1 during a 72 hour time window. The corresponding transcript profiles for ZIP10 and ZIP5 were more complex, with ZIP10 transcript levels undergoing a dramatic drop during the first few hours following EMT induction, but then continuously recovering, and eventually exceeding original levels. ZIP5 transcript levels, in contrast, were relatively low throughout EMT progression and did not exhibit a consistent trend-line. Having established that ZIP6 expression is induced during EMT, consistent with its potential involvement in this cellular program also in mammalian cell models, we next generated ZIP6 knockout clones by a 2^nd^ generation CRISPR/Cas9 knockout strategy (Fig. 1b). More specifically, NMuMG cells were co-transfected with an expression construct coding for point-mutated Cas9-D10A nickase and a pair of guide RNAs that directed the nickase to off-set target sites on opposite DNA strands within Exon 2 of the *Slc39a6* gene. Using this strategy, the successful generation of nearby nicks led to a double-strand genomic break, which triggered the cell-autonomous non-homologous end-joining (NHEJ) program. Following clonal isolation by the dilution method, a western blot-based screen identified several heterozygote and homozygote ZIP6 knockout clones. In this and subsequent experiments, the detection of ZIP6 relied on an in-house ZIP6-directed polyclonal antibody raised against a twelve amino acid ZIP6-specific sequence within the PrP-like ectodomain of this transporter, which was selected such that there would be no cross-reactivity with other ZIP family members (Fig. 1c). The subsequent western blot analysis of wild-type NMuMG cells and a ZIP6 knockout clone led to the expected increase in ZIP6 protein levels upon TGFB1 treatment in wild-type cells and confirmed the complete absence of this transporter in the ZIP6 knockout clone (Fig. 1d).

Genomic PCR and sequencing allowed us to identify a two nucleotide deletion in both *Slc39a6* alleles present in the NMuMG ZIP6 ko clone. The deletions generated a shift of the reading frame that caused the formation of premature STOP codons. In the course of these analyses we became aware of a hemozygotic deletion of an imperfect repeat sequence of 21 base pairs within Exon 1 in the wild-type *Slc39a6* sequence, which translates into an internal deletion of 7 amino acids for the expressed protein but is otherwise inconsequential for ZIP6 protein expression. We capitalized on this feature, which was retained in the ZIP6 ko clone during the characterization of the *Slc39a6* gene edits, as it allowed us to confidently determine that the same two nucleotide deletion had occurred in both alleles.

### ZIP6 forms a heteromeric complex with ZIP10 that interacts with NCAM1

**Figure 2:**
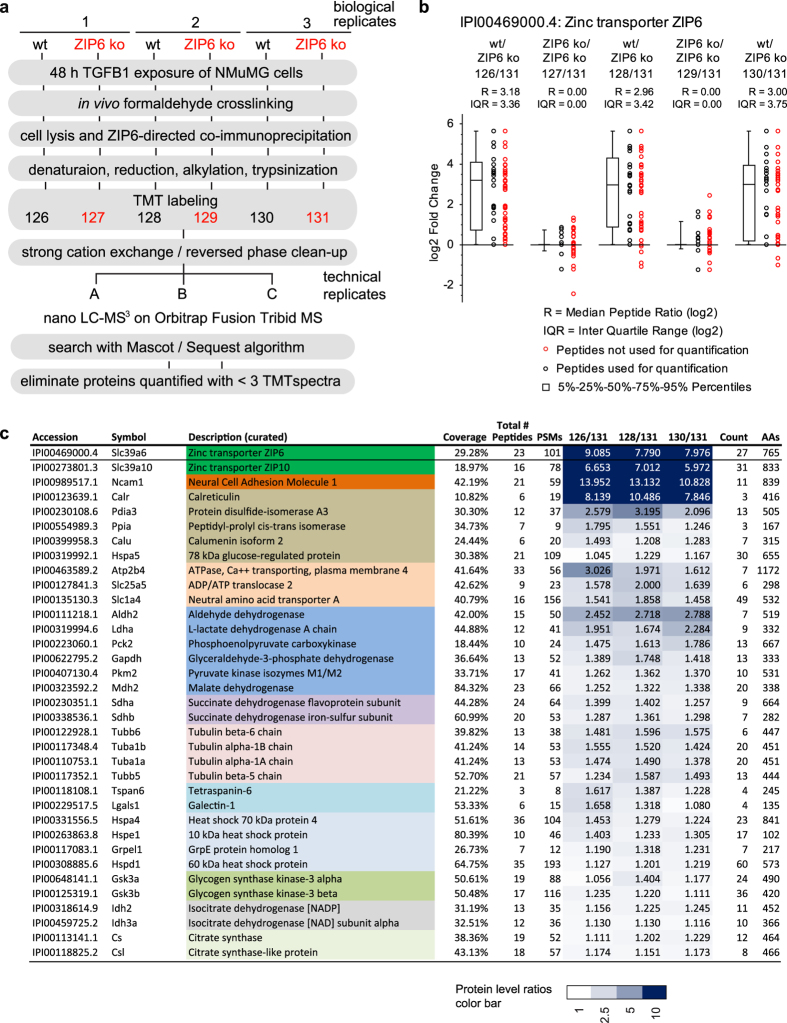
The ZIP6 interactome. (**a**) Chart depicting workflow of ZIP6 interactome analysis using wild-type or ZIP6 ko NMuMG cells as biological starting material. (**b**) Relative quantitation of ZIP6 levels in ZIP6 co-immunoprecipitations (co-IPs) from *in vivo* formaldehyde crosslinked wild-type and ZIP6 ko NMuMG cells in mass spectrometry dataset described in Panel ‘a’. Box plot depicting in log2 space enrichment ratios of individual ZIP6 peptides used for quantitation, as well as the median peptide ratio and Inter Quartile Range (IQR). A subset of peptides (indicated with red circles) was automatically eliminated from the quantitation because their identification was redundant or mass spectrometry profiles underlying their identification did not pass stringency thresholds. Note that in this and other box plots or tables in this paper relative protein levels are depicted as ratios, with ion intensities of the heaviest isobaric labels within multiplex analyses serving as the reference (denominator). (**c**) Truncated list of proteins enriched in ZIP6-specific co-IPs analyzed as described in Panel ‘a’. Colors identify proteins with related function and/or localization. The full table, including levels of these proteins in negative control samples, is shown in Supplementary Table S1.

**Figure 3:**
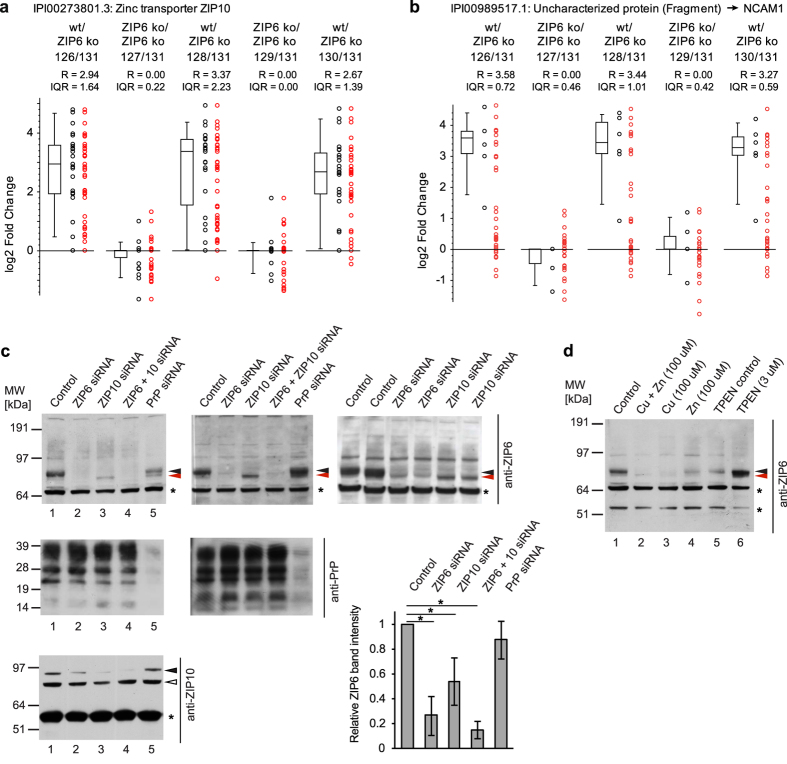
Interdependent assembly of a ZIP6-ZIP10 heteromeric complex that interacts with NCAM1. (**a**) Relative quantitation of endogenous ZIP10 levels in ZIP6 co-IPs from *in vivo* formaldehyde crosslinked wild-type and ZIP6 ko NMuMG cells. The box plot depicts in log2 space the enrichment ratios of individual ZIP10 peptides used for quantitation, as well as the median peptide ratio and Inter Quartile Range (IQR). See Fig. 2b for full legend and explanation of symbols. (**b**) Relative quantitation of NCAM1 in multiplex ZIP6 interactome dataset. (**c**) ZIP6 and ZIP10 interact and regulate each other’s expression also in Neuro 2a mouse neuroblastoma cells. Note the reproducible change in migration of ZIP6 bands upon knockdown of ZIP10, which may indicate an effect of ZIP10 on the post-translational modification or maturation of ZIP6 (the differences in band migration are demarked by red and black arrowheads). The graph depicts the results of densitometric analyses of band intensities (identified by filled arrow heads) from three independent analyses. Asterisks indicate cross-reactive bands. The open arrowhead indicates a band of uncertain identity. (**d**) Addition of copper or zinc to the cell culture medium of Neuro 2a cells mimics siRNA-based knockdown with regard to its effect on ZIP6 protein levels. In contrast, chelation of divalent cations by exposure of cells to TPEN causes an increase in steady-state ZIP6 protein levels. Because the treatment with TPEN required this chelator to be dissolved in ethanol, a separate negative control sample of cells exposed to an identical concentration of ethanol in the medium, designated as ‘TPEN control’, was included in the analysis.

We next employed wild-type NMuMG cells that had been treated with TGFB1 for 48 hours as biological source material to generate a first ZIP6 interactome dataset, with ZIP6 knockout NMuMG cells serving as negative controls (Fig. 2a). To avoid the inadvertent breaking of ZIP6 protein-protein interactions during cell lysis, cells were *in vivo* crosslinked by mild formaldehyde crosslinking prior to their harvest^3^. ZIP6 and proteins covalently crosslinked or loosely associated with it were subsequently co-immunoprecipitated with the aforementioned anti-ZIP6 antibody, denatured in urea and trypsinized. To minimize run-to-run variance and facilitate the direct relative quantitation of peptide levels, the analysis made use of a previously established work-flow that relies on the isobaric labeling of peptide mixtures with tandem mass tags (TMT) followed by the concomitant analyses of triplicate biological replicates and controls in a six-plex format^13^. Tandem mass spectrometry (MS/MS) analyses of peptide mixtures were undertaken on a Thermo Orbitrap Fusion instrument using a configuration for the sampling of TMT signature ion spectra and downstream data analysis we had previously reported^13^. Confirming successful and reproducible immunoprecipitation of ZIP6, the analysis unequivocally identified ZIP6 in the co-IP eluates derived from wild-type but not ZIP6 knockout cells (Fig. 2b and c, S1 Table) (note that well-known restrictions on the dynamic range of the quantitation method^15^ limited in this experimental design the ability to distinguish enrichment ratios that exceeded eight-fold). Interestingly, this analysis identified ZIP10 as a key interactor of the ZIP6 bait (Fig. 3a). NCAM1 (Fig. 3b) and calreticulin were the only two other proteins in the dataset whose detection appeared to strictly depend on ZIP6. However, several other proteins were also identified in ZIP6 co-IP eluates at levels that exceeded their quantities in negative controls, yet did not share the striking enrichment observed for the aforementioned binders. The latter distribution is characteristic of proteins that bind not only to the co-IP bait and its interactors but also to the affinity matrix itself, thereby leading to lower apparent TMT enrichment ratios. Pre-existing annotations of candidate ZIP6 interactors, which fell into this category identified most of them as belonging to relatively abundant cellular pathways or protein complexes, including (i) the endoplasmic reticulum-resident protein folding and quality control machine composed of calreticulin (CALR), calumenin (CALU), protein disulfide-isomerase A3 (PDIA3) and 78 kDa glucose-regulated protein (HSPA5), (ii) enzymes associated with the glycolytic pathway, (iii) subunits of microtubules, (iv) a subset of highly abundant heat shock / chaperone complex proteins, and (v) abundant dehydrogenase complex subunits. Additional ZIP6 interactors identified in this experiment, which shared relatively low enrichment levels but also cannot readily be classified as highly abundant proteins, were a small number of membrane-embedded transporters (ATP2B4, SLC25A5 and SLC1A4), tetraspanin-6 (TSPAN6), galectin-1 (LGALS1) and glycogen synthase kinase 3 alpha (GSK3A) and beta (GSK3B).

Several recent studies have revealed similarities and functional relationships between ZIP6 and ZIP10, suggesting that these two zinc transporters interact^16^,^17^,^18^. To further clarify their relationship, we investigated if reciprocal effects on expression and maturation of these proteins can be observed if the expression of one of them is transiently reduced. This experiment was undertaken in the mouse neuroblastoma Neuro2a cell model, which expresses both transporters at higher steady-state levels than NMuMG cells. Protein levels of ZIP6, ZIP10 and PrP were quantified by densitometry of Western blot signals (Fig. 3c). The experiment revealed a robust and significant reciprocal effect of ZIP6 and ZIP10 on each other’s expression, evidenced by a more than 50% reduction in protein levels and higher mobility—possibly reflecting incomplete maturation—of ZIP6 when ZIP10 expression was diminished by ZIP10-specific siRNAs. In contrast, siRNA-mediated depletion of PrP did not significantly affect total ZIP6 levels but also affected ZIP6 band migration, albeit in a less complete manner than ZIP10 depletion. To further explore the functional relationship of ZIP6 and ZIP10, we capitalized on our earlier observation that exposure of cells to elevated Cu^2+^ concentrations, rather than Zn^2+^, in the culture medium caused a pronounced reduction in ZIP10 protein levels^19^. If the expression and maturation of ZIP6 and ZIP10 proteins is co-regulated, we would expect a similar inhibition profile for ZIP6. Indeed, the presence of Cu^2+^ caused the same profound diminution of ZIP6 levels that we had previously observed for ZIP10 under identical experimental conditions (Fig. 3d). Moreover, as previously reported for ZIP10, chelation of divalent cations in the cell culture medium by the addition of N,N,N′,N′-Tetrakis (2-pyridylmethyl)ethylenediamine (TPEN) boosted steady-state levels of ZIP6. Taken together, these data are consistent with the interpretation that ZIP6 and ZIP10 form a functional heteromeric complex that interacts with NCAM1 and whose expression is tightly co-regulated, possibly involving critical interactions with calreticulin and other members of the ER-resident protein folding machinery.

### NCAM1 associates with focal adhesion complexes during EMT

**Figure 4:**
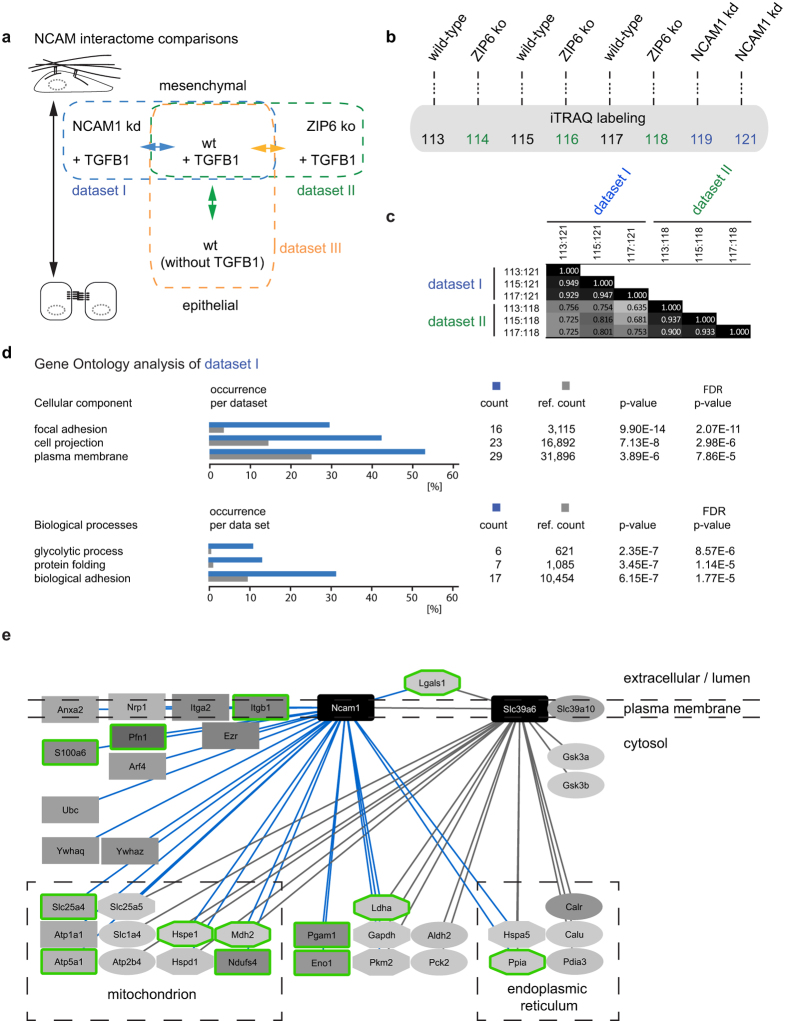
The NCAM1 interactome. (**a**) Design of three multi-plex NCAM1 comparative interactome analyses conducted in this study. Dataset I served the identification of NCAM1 interactors in TGFB1-treated NMuMG cells; Dataset II investigated the effect of ZIP6 ko on the NCAM1 interactome; and Dataset III studied the effect of epithelial-to-mesenchymal transition on the NCAM1 interactome. (**b**) The experimental design followed the workflow outlined in Fig. 2a. However, eight-plex iTRAQ labeling was employed to be able to generate Datasets I and II in one combined analysis. (**c**) Comparison of interactomes generated in Datasets I and II by Pearson correlation analysis. Note that biological replicates within Datasets I and II generated a Pearson coefficient near 1, indicating excellent biological and technical reproducibility of interactomes. A direct of comparison of Datasets I and II also returned Pearson coefficients >0.5, indicating a direct correlation, which suggests that the presence of ZIP6 promotes NCAM1 binding to a subset of its natural interactors. (**d**) Gene Ontology analysis of Dataset I. (**e**) Schematic depiction of ZIP6 and NCAM1 interactomes. The figure was originally generated in Cytoscape but nodes were subsequently rearranged to indicate the known predominant cellular compartments in which interactors reside. The intensity of grey shading correlates directly with the relative enrichment levels of interactors. Rectangular and oval node shapes identify NCAM1 and ZIP6 interactors, respectively. Octagonal shapes indicate the presence of a given interactor in both NCAM1 and ZIP6 interactome datasets. Nodes with green boundaries identify NCAM1 interactors whose presence in the NCAM1 interactome was most affected (>three-fold) by the presence or absence of ZIP6.

To better understand the relationship between ZIP6 and NCAM1, we next undertook three NCAM1 interactome analyses using essentially the same experimental design as outlined above for the ZIP6 interactome analysis (Fig. 2a). The three NCAM1 datasets investigated (i) the NCAM1 interactome in TGFB1-treated NMuMG cells with and without prior knockdown of NCAM1, thereby revealing binders of NCAM1 in this differentiation state (Dataset I), (ii) the influence of ZIP6 knockout on the NCAM1 interactome in TGFB1-treated NMuMG cells (Dataset II), and (iii) changes the NCAM1 interactome undergoes in cells shifting from an epithelial to a mesenchymal phenotype (Dataset III) (Fig. 4a). To facilitate the direct comparison of samples contributing to analyses I and II, we made use of an 8-plex labeling scheme based on isobaric tags for relative and absolute quantitation (iTRAQ)^20^ that afforded the ability to concomitantly obtain quantitation values for individual peptides from iTRAQ signature ions of three wild-type, three ZIP6 knockout and two NCAM1 knockdown samples (Fig. 4b). To gauge the reproducibility of biological replicates in this experiment, we computed the correlation coefficients of protein ratios by pair-wise comparisons of samples that gave rise to Datasets I and II (Fig. 4c). Indicative of high technical and biological reproducibility Pearson correlation coefficients consistently exceeded a value of 0.900 for pair-wise comparisons of biological replicates. However, Pearson correlation coefficients dropped to values between 0.635 and 0.816 in pair-wise comparisons of protein ratios observed in NCAM1 co-IP samples observed from Dataset I and II, suggesting that with regard to its influence on the NCAM1 interactome the ZIP6 knockout does not simply mimic an overall reduction of NCAM1 and its interactions (seen in Dataset I) but affects the NCAM1 interactome in a more complex way. Consistent with prior knowledge of NCAM1 biology, the subsequent gene ontology (GO) ‘Cellular Component’ analysis of Dataset I indicated that the NCAM1 interactome is highly significantly enriched in proteins that map to ‘focal adhesion’ complexes, ‘cell projections’ and the ‘plasma membrane’ (Fig. 4d). Perhaps less expected, the respective GO ‘Biological processes’ analysis of Dataset I indicated also a significant enrichment of proteins with known roles in ‘glycolytic processes’ and ‘protein folding’ (Supplementary Table S2). A meta-analysis of ZIP6 and NCAM1 interactomes generated in this work, which made use of Cytoscape software to analyze and highlight shared features, revealed considerable overlap but also differences in the composition of these two interactomes (Fig. 4e). More specifically, whereas the interactome of NCAM1 is consistent with its role as a hub protein, the ZIP6 interactome was much smaller and comprised only relative few interactors not shared by NCAM1. Notable exceptions to this trend were the exclusive interaction of GSK3A and B with ZIP6, as well as a more pronounced representation in the ZIP6 interactome of the aforementioned ER-resident proteins with known roles in protein folding and the quality control machinery.

### ZIP6 influences the association of NCAM1 with specific interactors

**Figure 5:**
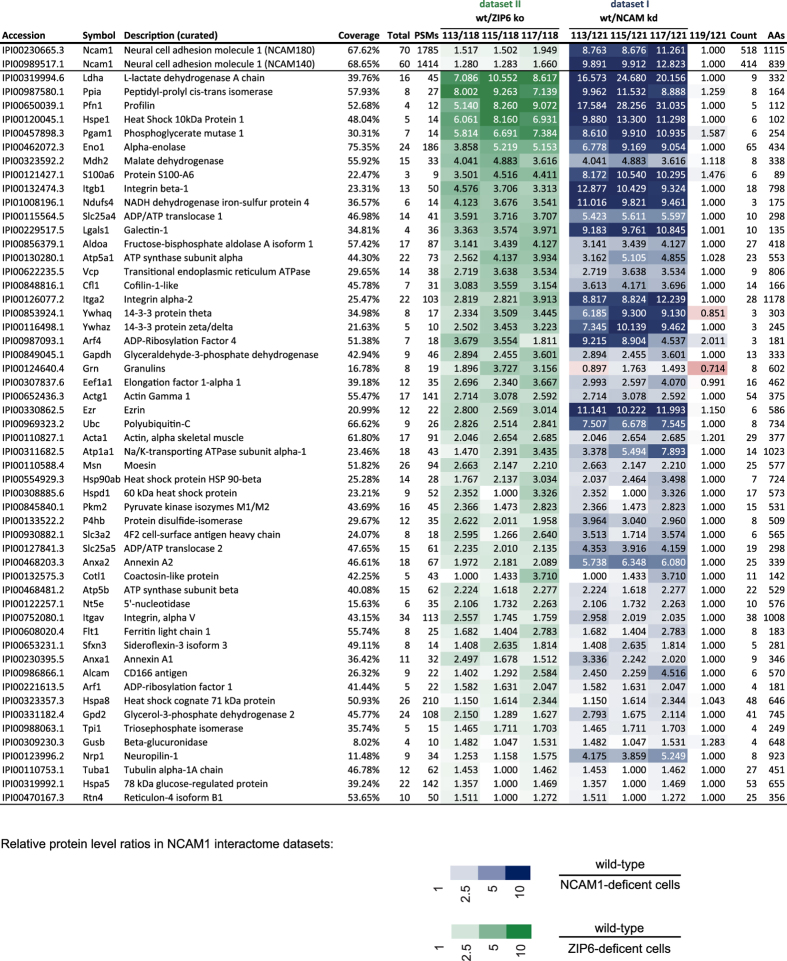
Effect of ZIP6 on the NCAM1 interactome. Subset of interactome data from Datasets I and II generated and labeled as described in Fig. 4a and b. NCAM1 interactors shown are sorted to reflect the influence of ZIP6 on the NCAM1 interactome, i.e., interactors, whose co-enrichment with NCAM1 was most affected in a direct comparison of NCAM1 interactome data generated from wild-type and ZIP6 ko, are listed first. Note that the Proteome Discoverer software used to compute the relative abundance ratios was configured to replace missing values in individual channels with minimum intensities observed. Because most NCAM1 interactors were absent from the negative control samples labeled with iTRAQ reagents 119 and 121, this configuration caused a majority of median iTRAQ 119/121 signature ion intensity ratios to give rise to a value of 1.

A close comparison of enrichment ratios of individual proteins in NCAM1 interactome Datasets I and II led to the following observations regarding the effect of ZIP6 knockout on NCAM1 and its interactors (Fig. 5): (i) In wild-type NMuMG cells, NCAM1 levels were approximately 50% higher than in ZIP6 knockout cells derived from them. (ii) The ZIP6 knockout affected primarily NCAM1 interactions with its key interactors (note the similar overall trend in shading intensity for protein ratios observed in Datasets I and II). More specifically, the ZIP6 knockout had a particularly pronounced effect on NCAM1 interactions with enzymes that play a broader role in glycolysis and the cellular energy household, as well as peptidyl prolyl cis-trans isomerase (PPIA), profilin (PFN1), heat shock 10 kDa protein 1 (HSPE1), protein S100-A6 (S100A6), and a subset of integrins. In contrast, interactions of NCAM1 with ezrin (EZR), polyubiquitin (UBC), annexin A2 (ANXA2), and neuropilin-1 (NRP1) were to a lesser degree affected by ZIP6 knockout than what would be expected if the deficiency of this ZIP transporter affected NCAM1 interactions observed in wild-type NMuMG cells equally.

### During EMT NCAM1 associates with integrins and actin-assembly complexes and is polysialylated in a manner that depends on PrP and is modulated by ZIP6

**Figure 6:**
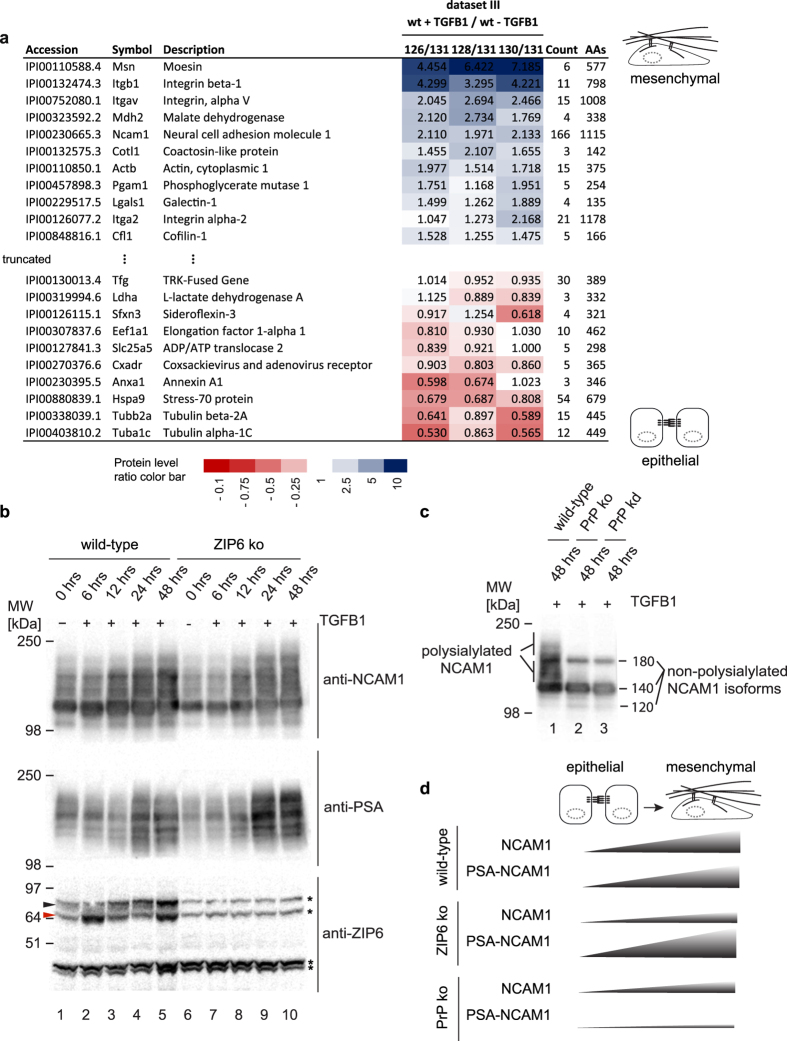
During EMT, a shift of NCAM1 cytoskeletal interactions from a tubulin-dominated to an actin-dominated molecular environment is accompanied by its polysialylation. (**a**) Relative quantitation of NCAM1 interactions before and after exposure of NMuMG cells to TGFB1. Changes in the relative levels of proteins which co-immunoprecipitated with NCAM1 are depicted as ratios. Red and blue background shading indicates proteins that bind to NCAM1 preferentially before and after the execution of EMT, respectively. (**b**) Time-course validation of changes in total NCAM1 levels and polysialylated NCAM1 during EMT in wt as well as ZIP6 ko cells. Note the partial overlap in the migration of ZIP6-specific (filled arrowheads) and crossreactive bands (indicated with asterisks). (**c**) Deficiency of PrP in NMuMG cells leads to loss of polySia modification on NCAM1. Western blot documenting ablation of NCAM1 polysialylation in PrP-deficient cells that underwent EMT. (**d**) Schematic depicting observed trends of relative levels of NCAM1 and polysialylated NCAM1 during EMT in wild-type, ZIP6-deficient, and PrP-deficient NMuMG cells.

We next investigated changes to NCAM1 interactions during EMT by generating the aforementioned NCAM1 interactome Dataset III (Fig. 4a) based on wild-type NMuMG cells before and after 48 hour exposure to TGFB1. The analysis was undertaken at a smaller scale than the NCAM1 interactome analyses underlying Datasets I and II and made use of six-plex TMT-tags for relative quantitation of three TGFB1-treated biological replicates and three vehicle-treated wild-type NMuMG negative control samples. The workflow for sample preparations, as well as mass spectrometry analyses and data processing followed the steps outlined before for generating the ZIP6 and NCAM1 interactome analyses. As expected for bait proteins in successful co-immunoprecipitation experiments, the NCAM1 bait again gave rise to the highest number of peptide-to-spectrum matches (PSMs), and its levels were observed to increase approximately two-fold during EMT (Fig. 6a). Interestingly, as NMuMG cells underwent EMT, interactions of NCAM1 with tubulin beta-2A (TUBB2A) and alpha-1C (TUBA1C)—presumably reflecting its interactions with the microtubule-based cytoskeleton—were reduced and replaced by interactions with the actin cytoskeleton. The latter was evident by consistently higher levels of actin (ACTB) itself, but also of moesin (MSN), integrins beta-1 (ITGB1), alpha V (ITGAV) and alpha-2 (ITGA2) and cofilin (CFL1) in the NCAM1 interactome from wild-type NMuMG cells of mesenchymal morphology, when compared to NMuMG cells of epithelial morphology. Of note, based on the relative quantitation results available for tubulin and actin, which are often observed as unspecific binders in interactome data sets, it can be concluded that their presence in specific eluates in this analysis represented to approximately equal parts NCAM1-dependent interactions and unspecific binding to the affinity matrix.

To assess if ZIP6 deficiency exerts a similar effect on NCAM1 polysialylation as PrP deficiency^13^, we next harvested wild-type and ZIP6 knockout NMuMG cells at distinct time intervals following the addition of TGFB1 to the cell culture medium and analyzed cell lysates by Western blotting with antibodies directed against total NCAM1 or its polysialic acid (polySia) modification (Fig. 6b). This analysis revealed that ZIP6 deficiency reduced the overall amount of NCAM1 in NMuMG cells but, unlike PrP deficiency (Fig. 6c), did not prevent NCAM1 polysialylation. In contrast, despite reduced overall NCAM1 protein levels in ZIP6 knockout cells, the intensity of polySia bands was increased in the absence of ZIP6. Taken together, these data suggest that the presence of ZIP6 exerts, like the presence of PrP^13^, a positive effect on total NCAM1 levels but, whereas PrP deficiency abolishes NCAM1 polysialylation in this model, ZIP6 deficiency promotes it (Fig. 6d).

### Phosphorylation of NCAM1 at a GSK3 consensus site

**Figure 7:**
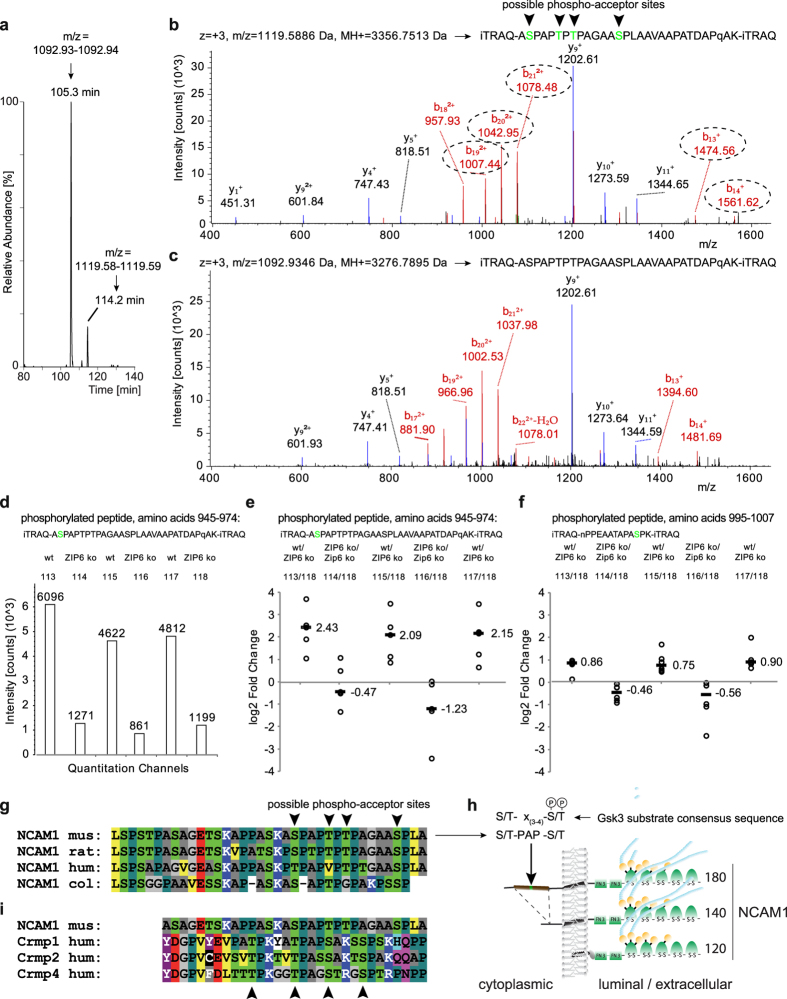
ZIP6 controls phosphorylation of the longest isoform of NCAM1 at a phospho-acceptor site that conforms to a previously described GSK3 recognition site within members of the Crmp protein family. (**a**) Extracted ion chromatograms of a specific NCAM1 peptide and its phosphorylated derivative that were repeatedly identified and quantified in Datasets I and II. The relative peak intensities suggest that approximately 15% of the peptide was phosphorylated. Panels (b) and (c) depict tandem mass spectra underlying the identification of phosphorylated and non-phosphorylated NCAM1 peptide, respectively. Note the characteristic m/z shift observed for a subset of fragment ions of the b-series (highlighted in dotted ovals), which identify the phosphorylation. (**d**) Phosphorylation of NCAM1 at the specific site depends on the presence or absence of ZIP6. iTRAQ signature ion trace of tandem mass spectrum depicted in panel ‘c’. (**e**) Plot depicting the enrichment ratios of repeated analyses of the phosphopeptide depicted in panel ‘c’, documenting four-fold higher phospho-occupancy levels for this peptide in NCAM1 co-IPs from wild-type cells than in ZIP6 ko cells. (**f**) Relative levels of an unrelated NCAM1 phosphopeptide that may serve as an internal negative control, because its phospho-occupancy levels are to a lesser degree dependent on ZIP6 than those of the phosphopeptide quantified in panel (e). Note that the two-fold higher levels of the phosphopeptide in wild-type over ZIP6 ko cells only marginally exceed the relative ratios of non-phosphorylated NCAM1-derived peptides, which were also observed at approximately 1.5-fold higher levels in wild-type than in ZIP6 ko cells (see Fig. 5). (**g**) Multiple alignment of NCAM1 sequence stretch encompassing the ZIP6-dependent phosphorylation site. The sequence stretch comprises a cluster of candidate phospho-acceptor sites that conform to a GSK3 substrate consensus motif, depicted in (**h**), which maps to a cytoplasmic insertion coded by Exon 18 present only in the longest NCAM1 isoform. (**i**) The ZIP6 dependent NCAM1 phosphorylation site bears striking resemblance to a previously reported GSK3-phosphorylation site present in a subset of Crmp proteins.

Because Dataset II led to 1,785 peptide-to-spectrum matches, which were assigned to the longest isoform of NCAM1, cumulatively covering 68% of its sequence, it afforded the identification and relative quantitation of several NCAM1 post-translational modifications. One of them was a phosphorylation on the NCAM1 peptide (residues 945–974) with the amino acid sequence ‘ASPAPTPTPAGAASPLAAVAAPATDAPQAK’, which was repeatedly assigned to more than a dozen independent tandem mass spectra both in its phosphorylated and unphosphorylated state (Fig. 7a–c). Intriguingly, in contrast to other NCAM1 phosphorylation sites observed in the dataset, including a peptide which maps to a nearby stretch of amino acids with the sequence NPPEAATAPASPK (residues 995-1007), phosphorylation of the 945-974 peptide within NCAM1 was profoundly dependent on the presence of ZIP6 (Fig. 7d–f). The peptide harbors several phospho-acceptor sites, of which as many as four might have been phosphorylated (there was some ambiguity due to gaps in the y- and b-ion series of backbone fragments). Closer inspection of the respective sites characterizes them as being two, four and six residues apart and embedded within an amino acid sequence stretch rich in prolines and alanines that also comprises two lysine residues and appear in NCAM-180 only due to the inclusion of Exon 18 in this longest NCAM1 isoform (Fig. 7g). Clusters of nearby phosphorylation sites are also known in other proteins. Amongst the known kinases, GSK3A and B are especially well-known for their ability to transfer phosphates to sites of this nature, in particular, once a priming phosphate has been attached to the most C-terminal acceptor site within such a cluster. We therefore compared the NCAM1 phosphorylation cluster both to the GSK3 substrate consensus sequence S/T-x_(3-4)_-S(P)/T(P)^21^ (Fig. 7h) and to validated GSK3 substrates. Surprisingly, a known GSK3 substrate sequence within the family of collapsin response mediator proteins^22^ showed striking similarity in both its overall amino acid composition and sequence to the NCAM1 cluster identified in this work, sharing with NCAM1 the core sequence A(S/T)PAP(S/T) (Fig. 7i). Taken together, these data strongly implied a model whereby GSK3 kinases contribute to NCAM1 phosphorylation within this cluster, and the aforementioned co-enrichment of GSK3A and B with ZIP6 accounts for the ZIP6-dependence of the observed phospho-occupancy at this site.

### *In vitro* phosphorylation of the longest NCAM1 isoform by GSK3B

**Figure 8:**
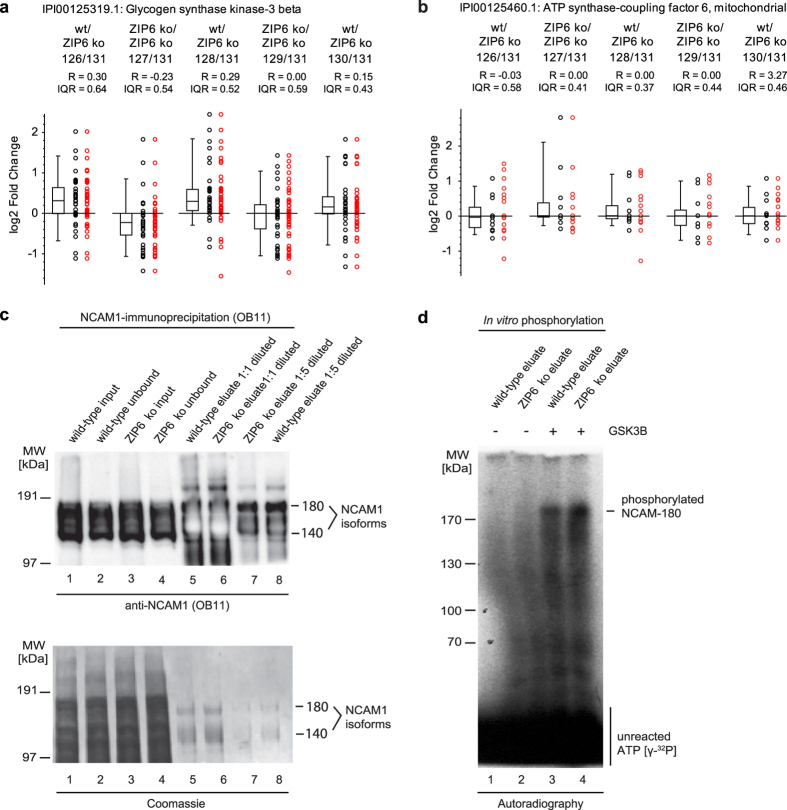
*In vitro* phosphorylation of longest NCAM1 isoform by GSK3B. (**a** and **b**) Box plots showing relative quantitations of glycogen synthase kinase 3 beta (GSK3B) and ATP synthase coupling factor 6 (ASCF6) in NCAM1 interactomes generated with wild-type or ZIP6 ko cells. Whereas GSK3B protein levels in the NCAM1 interactome correlated directly with the presence or absence of ZIP6, ASCF6 protein level ratios were similar in all samples, consistent with unspecific binding of this protein to the affinity capture matrix. (**c** and **d**) Evidence of NCAM-180 *in vitro* phosphorylation by GSK3B. (c) Immunoprecipitation of NCAM1 from cellular extracts of NMuMG cells, which had been induced to undergo EMT by 48 h addition of TGFB1 to the cell culture medium. The OB11 antibody used in this experiment for immunoprecipitation and Western blot detection binds to a cytosolic epitope shared by NCAM-140 and NCAM-180, which gave rise to the characteristic band pattern in Lanes 5 and 6. Note that due to its relatively higher abundance in eluate fractions, signals for NCAM-140 exceeded maximum intensity levels in lanes 5 and 6, leading to a partially inverted ‘white’ signal. (d) Autoradiographic analysis of *in vitro* GSK3B phosphorylation of NCAM1 immunoprecipitation eluates seen in Panel ‘c’. Note that although the signal detected at 180 kDa, corresponding to NCAM-180, has not been validated to consist of phosphorylated NCAM1, the Coomassie stain of the NCAM1 IP eluate fractions shown in Panel ‘c’ revealed no signals in the high mass region of the gel that could not be attributed to the expected NCAM-180 or NCAM-140 bands.

Observations thus far raised the intriguing possibility that the ZIP6-ZIP10 heteromer may serve as a scaffold for binding and directing GSK3 kinases to their NCAM1 substrate. A close look at the evidence for ZIP6 binding to GSK3A and B assured us that this conclusion was robust. Note that the ability to confidently assign even small differences in relative abundance levels increases in this methodology with the statistical power inherent to the number of peptides quantified and matched to a given protein. Thus, although GSK3A and GSK3B levels in ZIP6-specific co-IP eluates exceeded in this work the levels of these proteins in the negative control sample by only ~20% (Fig. 8a), this difference was significant and distinct from the sample distribution of, for example, ATP synthase coupling factor 6 (ATP5J), a mitochondrial protein, which was observed at equal levels in samples and negative controls, presumably, because it bound to the affinity matrix exclusively through non-specific interactions (Fig. 8b).

To validate whether the longest NCAM1 isoform is indeed a GSK3 substrate, we next tested if recombinant GSK3B can phosphorylate NCAM1 that has been immunoprecipitated from NMuMG cells following 48 h induction of EMT by TGFB1. This design was selected over an alternative strategy, whereby both the kinase and putative substrate would be obtained from recombinant sources, because GSK3 typically requires a priming phosphorylation that would not be present in recombinant NCAM1. Corroborating the mass spectrometry results, this experiment revealed that a 180 kDa protein, corresponding in size to the longest isoform of NCAM1, was indeed the prime phosphorylation target of recombinant GSK3B in the NCAM1-directed immunoprecipitation eluate (Fig. 8c and d). Taken together, these data provided orthogonal evidence for the notion that GSK3B can preferentially phosphorylate the longest NCAM1 isoform in biological material relevant to this study.

### A histidine-rich cytoplasmic loop mediates binding of GSK3B to the ZIP6-ZIP10 heteromer

**Figure 9:**
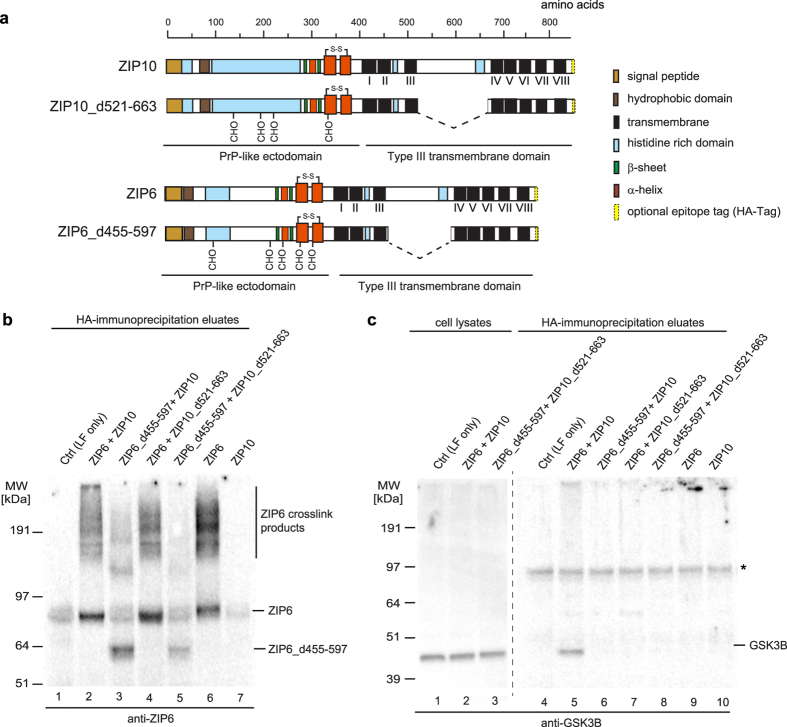
GSK3 binds to scaffold formed by histidine-rich cytoplasmic loop domains within the ZIP6-ZIP10 heteromer. (**a**) Domain organization of ZIP10 and ZIP6 and deletion constructs used in this work to test the hypothesis that a cytoplasmic loop connecting transmembrane (TM) domains III and IV is essential for binding to GSK3 binding. (**b**) Control Western blot documenting ZIP6 expression products in eluates shown in panel ‘e’. (**c**) The co-expression of wild-type ZIP6 and ZIP10 proteins comprising intact cytoplasmic loop domains connecting TM domains III and IV is essential for binding of GSK3. GSK3-directed Western blot analysis of cellular lysates and co-immunoprecipitation eluates from *in vivo* formaldehyde crosslinked cells expressing different combinations of wild-type and/or mutant ZIP6/ZIP10. Note that in addition to protein bands reflecting the expected monomeric molecular weights of GSK3 and ZIP6, the Western blots in panels ‘d’ and ‘e’ revealed the appearance of high molecular weight crosslinked bands, whose appearance depended on the application of the *in vivo* formaldehyde crosslinking step.

Both ZIP6 and ZIP10 are composed of large N-terminal ectodomains and C-terminal segments that comprise eight transmembrane domains. Except for one cytoplasmic loop connecting transmembrane domains III and IV, the membrane topology of these divalent metal ion transporters creates very little cytoplasmic surface that could engage in interactions with cytoplasmic binding partners, such as GSK3 kinases. To validate the interaction and determine if this cytoplasmic loop domain is indeed critical for the interaction of ZIP6 and/or ZIP10 with GSK3 kinases, we generated internal deletions of the respective loop domains within eukaryotic ZIP6 and ZIP10 expression plasmids carrying C-terminal hemagglutinin (HA) affinity capture tags (Fig. 9a), transfected them into NMuMG cells and induced EMT by the addition of TGFB1. Following *in vivo* formaldehyde crosslinking, protein complexes containing ZIP6 and/or ZIP10 were co-immunoprecipitated with HA-directed affinity matrices and eluate fractions analyzed by Western blotting. The analysis with a ZIP6-directed antibody verified the successful transfection and expression of the intended wild-type and internal deletion constructs (Fig. 9b). Probing of cell lysates and eluate fractions with a GSK3-directed antibody led to the detection of a 45 kDa signal, consistent with the expected molecular mass of GSK3B (Fig. 9c). Remarkably, only the co-expression of wild-type ZIP6 and ZIP10 caused the appearance of a band with an identical molecular mass in the eluate fraction. Thus, neither the expression of ZIP6 or ZIP10 alone, nor the expression of any combination of ZIP6-ZIP10 heteromer containing an expression product with an internal deletion was observed to co-immunoprecipitate GSK3, consistent with the interpretation that its binding may involve components of the cytoplasmic loop domains from both ZIP zinc transporters.

## Discussion

This study set out to undertake a hypothesis-free investigation of the ZIP6 interactome in a widely used mammalian cell model for studying EMT. It uncovered that ZIP6 forms a heteromeric complex with ZIP10 that predominantly interacts with NCAM1. This discovery triggered a 2^nd^ set of interactome analyses, which first produced an in-depth analysis of the molecular environment of NCAM1 in the same cell model and, next, explored how the knockout of ZIP6 affects NCAM1 interactions. The study established prominent interactions of NCAM1 with integrins that are modulated by ZIP6, thereby corroborating observations by others, which had previously documented that NCAM1 (i) either associates directly with focal adhesion complexes during EMT in the NMuMG model^23^, or (ii) induced focal adhesions in a cell-cell contact dependent manner in a human neuroblastoma cell model^24^. We documented that ZIP6—like its PrP cousin—promotes an increase in steady-state NCAM1 levels during EMT but—unlike PrP—serves to limit NCAM1 polysialylation that occurs during the execution of this morphogenetic program. Following up on the observation of a ZIP6-dependent phosphorylation of a specific NCAM1 peptide, we identified a cluster of phospho-acceptor sites in the cytoplasmic domain of the longest isoform of NCAM1, which conforms in its composition and core sequence to a known GSK3 substrate cluster within the family of collapsin-response mediator proteins. We then went on to show by *in vitro* phosphorylation assay that NCAM-180 can indeed serve as a GSK3B substrate. Finally, we provided a mechanistic explanation for this ZIP6-dependent NCAM1 phosphorylation by showing that cellular GSK3 is recruited to a novel binding site for this kinase that involves histidine-rich cytoplasmic loops present in the Type III transmembrane domains of the ZIP6-ZIP10 heteromer.

The ZIP6 interactome dataset generated in this work may represent the first investigation of molecular interactions of any ZIP metal ion transporter that employed a mass spectrometry-based discovery workflow. It has been known for some time that ZIP6 and ZIP10 co-purify under certain conditions^3^, are co-regulated in some paradigms^16^,^17^,^25^, and share functional similarities^17^. The functional relationship between these ZIP transporters was particularly evident in the latter study, which documented that during meiotic maturation transcript levels of ZIP6 and ZIP10 were correlated and exceeded transcript levels of other zinc transporters six- to tenfold. The authors went on to show that the targeted disruption of maternally-derived ZIP6 or ZIP10 during meiotic maturation perturbed intracellular zinc levels and resulted in cell cycle arrest. The first evidence that ZIP6 and ZIP10 may, in fact, bind directly to each other emerged from a functional analysis of ZIP10 in the zebrafish gastrulation paradigm^18^. In this recent work, the ZIP10 knockdown not only mimicked a previously reported gastrulation arrest phenotype observed with ZIP6-deficient zebrafish embryos^9^ but the two proteins were seen to interact intimately on the basis of proximity ligation assay and co-immunoprecipitation analyses. The current study corroborated these prior observations and added to this body of research by showing that in the cell models employed in this work (i) ZIP6 interacts with ZIP10 but not with other ZIP transporters, (ii) ZIP6 and ZIP10 are not only co-regulated but influence each other’s expression reciprocally, a property often observed amongst subunits of functional protein complexes, and (iii) ZIP6 and ZIP10 share a rapid suppression of their steady-state protein levels in cells exposed to increased copper concentrations. Currently unanswered questions remain regarding the precise stoichiometry of the ZIP6/ZIP10 heteromer and the spatiotemporal dynamics of its assembly. Rather than exhibiting perfect co-localization, our preliminary data in neuroblastoma (Neuro2a) cells suggest that the cellular distribution of ZIP6 and ZIP10 is only partially overlapping (unpublished results), consistent with a model that sees them cooperate only in specific cellular contexts and paradigms.

A central finding of this study was the highly reproducible and selective enrichment of NCAM1 in ZIP6 co-IPs. To our knowledge, NCAM1 had not been described to associate with any ZIP metal ion transporter before. Yet, this discovery was not surprising for anyone aware of both (i) the evolutionary relationship of ZIP zinc transporters and PrP, and (ii) the fact that NCAM1 has repeatedly been shown to not only interact with PrP^14^,^26^,^27^,^28^,^29^ but represented the most prominent *in vivo* crosslinking partner of PrP in the neuroblastoma cell model^14^. The latter study not only provided evidence for a direct interaction of PrP and NCAM1 but also identified on the basis of *in vitro* binding data a tentative binding interface that encompassed Helix A and the adjacent loop domain within PrP and β-strands C and C’ in the two fibronectin type III (FNIII) domains within NCAM1. In light of the predicted structural similarity between PrP and ectodomains of ZIP5, 6 and 10^7^,^30^ one may speculate that the interaction between the ZIP6/ZIP10 heteromer involves a homologous binding interface. If so, it would seem plausible that binding of the ZIP6/ZIP10 heteromer could compete with binding of NCAM1 to other cell surface proteins that interact through interfaces involving FNIII domains of NCAM1. A candidate would be the FGF receptor, which is not only understood to use such a binding interface^31^ but is also displaced during EMT, coinciding with the expression of ZIP6^23^. Consistent with the view that ZIP6 is not merely a bystander in NCAM1-related cell biology, ZIP6 knockout cells did not only express lower levels of NCAM1 but were observed to exhibit profound and reproducible impairments in NCAM1 interactions during EMT in this study. In particular, the ability of NCAM1 to interact with glycolytic enzymes, components of the actin cytoskeleton, integrins and 14-3-3 proteins was impacted in ZIP6 knockout cells. Naturally, because a majority of these interactions could not be further investigated within the scope of this study, they should be considered tentative until validated with orthogonal methods. It will be of particular interest to resolve if the ZIP6-ZIP10 heteromer affects these NCAM1 interactions in a manner that is dependent on the divalent cation import capabilities of these ZIP transporters or operates independently of them.

Two independent observations placed a spotlight on GSK3 kinases in this work: First, the ZIP6-ZIP10 heteromer was observed to co-immunoprecipitate with both GSK3 paralogs. Second, we discovered a ZIP6-dependent phosphorylation site within the cytoplasmic domain of the longest isoform of NCAM1 that bears striking similarities to a previously known GSK3 substrate. Our data place GSK3 paralogs in close proximity to the site of ZIP6-ZIP10-mediated zinc influx—presumably one of only few cytoplasmic locations at which free zinc ion concentrations may exceed the generally very low levels of free zinc in the cytoplasm. This observation may be relevant in light of prior work by others which documented that the ability of GSK3 to phosphorylate its substrates is inhibited in the presence of free zinc^32^. The authors documented that there is specificity to this zinc-mediated GSK3 inhibition by showing its reliance on zinc, as opposed to other divalent cations, and by establishing that the zinc inhibition characteristic does not extend to CDK2, a closely related kinase. Putative sites for GSK3 binding to the ZIP6-ZIP10 heteromer were mapped in this work to a cytoplasmic loop connecting transmembrane domains III and IV (amino acids Fig. 9 597 and 521-663 in ZIP6 and ZIP10, respectively). Similar to known docking sites of GSK3 on axin 1 (AXIN1) and 2 (AXIN2)^33^,^34^, these cytoplasmic loop segments within the two ZIP transporters are enriched in charged residues and C-terminally flanked by a histidine-rich segment. It has long been known that NCAM1 can be phosphorylated within its intracellular domain by casein kinase 1 (CK1) and GSK3 but in these early studies the phospho-acceptor site was not determined^35^. Subsequent attempts to map the NCAM1 phosphorylation sites highlighted initially a domain in juxtaposition to the inner face of the plasma membrane but also a putative serine 761 phospho-acceptor site that conforms to the GSK3 consensus motif^36^. The design of the latter phospho-site mapping experiments precluded the detection of phosphorylation sites observed in this study. This was because analyses were limited to a truncated NCAM1 expression construct comprising the transmembrane and cytoplasmic domains of NCAM1 but lacking the Exon 18 coded alternatively spliced insertion that is only present in the longest NCAM1 isoform. However, a more recent global phospho-site analysis of developing mouse brain samples independently mapped amino acids 946 and 958 (NCBI accession number: NP_001106675) as NCAM1 phospho-acceptor sites^37^. These sites are identical to the first and fourth NCAM1 phosphorylation sites within the phosphorylation cluster (amino acids 945-974) that was repeatedly sequenced and quantified in this study. We subsequently validated the preferential GSK3B phosphorylation of NCAM-180 by *in vitro* phosphorylation assay, using immunoprecipitated NCAM-140 and NCAM-180 as substrates. Curiously, CRMP2, a member of the CRMP family, which is known to carry a highly similar GSK3 phosphorylation motif (Fig. 7i), had previously also been shown to be a binder of NCAM1^38^, but was not detected in the interactome dataset produced in this study.

What might be the functional consequences of the GSK3-dependent phosphorylation of NCAM1? One commonly observed scenario would see phospho-occupancy at this site alter NCAM1 interactions with phospho-serine/threonine (pSer/Thr) binding modules on other proteins. In fact, binding of NCAM1 to 14-3-3 proteins, the first signaling molecules recognized to engage in pSer/Thr-dependent interactions with other proteins, was also observed to be ZIP6-dependent in this study, making them excellent candidates for this scenario. While the broader physiological consequences are not known, a previous report established that inhibition of GSK3 can prevent NCAM1-induced neurite outgrowth^39^.

In conclusion, the discovery of the evolutionary relationship between ZIP transporters and PrP a few years ago^7^ raised the possibility that the study of ZIP transporters may provide a promising angle for uncovering the elusive function of PrP. Indeed, similarities in gastrulation defects in ZIP6- or PrP-deficient zebrafish embryos precipitated a productive line of research, which revealed that PrP modulates the expression of NCAM1 and controls its polysialylation during EMT^13^. Observations in this study remind us that relationships can work in more than one direction. Prior knowledge of NCAM1 as the key interactor of PrP in several paradigms placed the discovery of NCAM1 as the most prominent ZIP6 interactor in a special light. Foremost, it suggests that PrP inherited its NCAM1 binding properties from its ZIP ancestor. This conclusion is consistent with bioinformatic evidence, which indicates that NCAM and ZIP transporters carrying a PrP-like ectodomain go back in evolution approximately one billion years but the emergence of the prion gene family and polysialyltransferases required for NCAM1 polysialylation represent more recent evolutionary inventions that occurred early during vertebrate speciation^40^. Our data reveal the existence of an intricate relationship that sees ZIP6 control the expression, post-translational modification and interactions of NCAM1 in the context of larger morphogenetic rearrangements executed at various stages during development and in cancers. It is tempting to speculate that the prion gene owes its survival to an adaptation that allowed it to modulate the same overall NCAM1-centric biology but provided a fitness advantage in the form of its control of NCAM1 polysialylation.

## Experimental Protocol

### Antibodies and siRNAs

For immunoblotting, primary antibodies for ZIP6 and ZIP10 (generated in-house)^19^, PrP (1:2000, A03213; Bertin Pharma, France), HA.11 (901501; BioLegend, CA, USA), NCAM1 (1:6666, 556324; BD Biosciences, ON, Canada), PSA-NCAM1 (1:1000, 556324; BD Biosciences), the cytosolic domain of NCAM1 (C9672, clone NCAM-OB11, Sigma-Aldrich, ON, Canada) and GSK3 (1:1000, AF2157; R&D Systems, MN, USA) were used. For transient knockdowns, SilencerSelect siRNAs (cat. no. 4390771) targeting transcripts of *Slc39a6* (s98749), *Slc39a10* (s105558), *Prnp* (s72188) and *Ncam1* (s70398) were sourced from Life Technologies (Carlsbad, CA, USA).

### Cell culture and transfection

Mouse mammary gland NMuMG cells (CRL-1636) were a kind gift from Dr. Jeffrey Wrana (University of Toronto, ON, Canada), but are also available commercially through the American Type Culture Collection (ATCC) (Manassas, VA, USA). Mouse neuroblastoma Neuro-2a (N2a) (CCL-131) cells were purchased through the ATCC. Cells were cultured as recommended by the ATCC, with 10% heat inactivated FBS (12484028; Life Technologies), 1% GlutaMAX (35050061; Life Technologies), and 1% antibiotic-antimycotic solution (15240062; Life Technologies) in (Dulbecco’s) Modified Eagle medium. Human insulin solution (I9278; Sigma-Aldrich, ON, Canada) was also added at a concentration of 10 μg/mL for NMuMG cells. To transfect cells with siRNAs, Lipofectamine RNAiMAX (13778075; Life Technologies) was used according to the manufacturer’s instructions. The treatment of cells with divalent cations or the cell-permeable chelator TPEN (P4413, Sigma-Aldrich) occurred for a duration of 48 h and was undertaken as described before^19^.

### Generation of *Slc39a6* CRISPR-knockout clones

The gRNA expression plasmid MLM3636 (Plasmid 43860) and SpCas9 plasmid JDS246 (Plasmid 43861) were obtained from Addgene’s non-profit plasmid repository (Cambridge, MA, USA). The SpCas9nD10A nickase plasmid, provided by Louisa Wang, was generated by site-directed mutagenesis of the original JDS246 plasmid using a Q5 Site-Directed Mutagenesis Kit (E0554S; New England Biolabs, Ipswich, MA, USA) and forward and reverse primers AGTGCCGATGGCTAAACCAATAG and AATTCCGTTGGATGGGCTG, respectively. Compatible CRISPR nickase target sites within *Slc39a6* Exon 2 were identified using the online ‘CRISPR Design Tool’ (http://crispr.mit.edu/). Respective oligonucleotide pairs were subsequently customized and integrated into the MLM3636 plasmid as described in a previous report^12^. NMuMG cells were transfected using Lipofectamine LTX (15338100; Life Technologies) and a 6:1:1 (w/w/w) mixture of SpCas9nD10A and customized gRNA plasmid pairs. Transfections were completed according to the manufacturer’s instructions at a cell confluency of approximately 75%. 48 hours post transfection, cells were harvested, diluted to a concentration of 1 cell/mL of culture medium, and replated. After one week, individual colonies were picked and cultured in separate wells of a 24-well plate before being transferred to 6-well and then 60 mm plates, when cell numbers became sufficient for Western blot analyses.

### Genomic PCR analysis of *Slc39a6* ZIP6 knockout cells

Genomic PCR on wildtype and CRISPR-Cas9-edited NMuMG cells was performed using the Q5 Hot Start High-fidelity DNA polymerase master mix (catalog number M0494S, Ipswitch, New England Biolabs, Massachusetts, USA) on 25 ng of extracted genomic DNA (catalog number K182001, PureLink Genomic DNA Mini Kit, Invitrogen, Carlsbad, CA, USA) at an annealing temperature of 67 °C for 35 cycles. Two separate pairs of primers flanking the CRISPR-edited region were used to assess and confirm editing specificity. Sequences for primer Pair 1 are TGACCTTTGCCCTTTGGGTT and ATCTTTGGTGCGTCTCCCAG. Sequences for primer Pair 2 were CACAAGCCCGTAGTCAGTCA and GATGCTGGGTGGGGTAGAAG. PCR products were purified (catalog number 28106, QIAquick PCR Purification Kit, Qiagen, Toronto, ON, Canada) before being sent for sequencing.

### Cloning of expression vectors coding for ZIP6 and ZIP10 with internal deletions

The cloning of mouse ZIP6 and ZIP10 expression constructs with internal deletions was based on parental expression plasmids we had described before^19^. The site-directed mutagenesis made use of HiFidelity polymerase (Qiagen, Valencia, CA, USA) and was designed to cause the deletion of residues 455–597 within mouse ZIP6 (IPI00469000.4, comprising CDS base pairs 2,589 to 3,017) and amino acids 521–663 within mouse ZIP10 (IPI0273801.3, corresponding to CDS base pairs 4,972 to 5,400), respectively. Final clones were sequence verified.

### Western blot analyses

Cells were grown to near confluency, growth medium was removed, and cells were rinsed twice with ice-cold phosphate buffered saline (PBS) before being lysed in a buffer consisting of 50 mM Tris (pH 8.0), 150 mM NaCl, 1% NP-40, and 1× Complete Protease Inhibitor Cocktail (11836170001; Roche, ON, Canada). Cellular debris were cleared by centrifugation for 5 minutes at 14 000 RPM and 4 °C, and protein levels were subsequently adjusted using a bicinchoninic acid (BCA) colorimetric assay. Equal amounts of protein were separated on 4–12% or 12% Bis-Tris (Life Technologies) or 7% Tri-Glycine (cast in-house) gels before being transferred to a 0.45 micron polyvinylidene fluoride (PVDF) membrane. The blot membranes were blocked with 10% skim milk in Tris-buffered saline and Tween 20 (TBST), and incubated overnight at 4 °C with the respective primary antibody, diluted in 5% skim milk in TBST. The blots were washed thrice with TBST and were incubated with HRP-conjugated anti-mouse (1:5000, 170–6516; BioRad), anti-rabbit (1:5000, 170–6515; BioRad), or anti-rat (1:5000, 31476; Thermo Fisher Scientific, Waltham, MA, USA) secondary antibodies diluted in 5% skim milk in TBST for 1–2 hrs at RT. Blots were again washed thrice with TBST before being briefly incubated with the ECL reagent (RPN2106; GE Healthcare). Signals were then visualized using either X-ray film or a LI-COR Odyssey Fc digital imaging system (LI-COR Biosciences, NE, USA).

### RT-PCR

Real-time polymerize chain reaction was completed identically to the procedure described in a recent report^13^, with the exception that the mRNAs targeted were specific to *Slc39a5* (Mm00511105_m1), *Slc39a6* (Mm00507297_m1), and *Slc39a10* (Mm00554174_m1). In brief, qPCR analyses were based on three technical replicates representing independent amplification reactions. Note that the specific primers employed are not known to us because the mRNA expression analyses were undertaken with TaqMan assays for which the providers do not share proprietary information on primers or FAM-MGB probes. However, the *Slc39a5* qPCR assay is designed to target RefSeq NM_001136237.1 to generate a 75 bases amplicon mapping to Exon boundary 4–5; the *Slc39a6* assay is based on primers targeting Exon boundary 8–9 within RefSeq entry NM_139143.3; and the *Slc39a10* assay targets a 70 bases amplicon at the Exon boundary 4–5 of RefSeq sequence NM_172653.2.

The computational analysis and relative quantification of qPCR data was based on the qBASE PLUS software (Biogazelle NV, Belgium) using the ddCt method after normalization to *Hprt* and *Tfrc* mRNAs. The latter gene transcripts were selected on the basis that their levels were observed to be least affected during the course of the TGB1 treatment, when compared to other housekeeping genes we tested. The relative expressions of target transcripts were scaled to samples derived from vehicle-treated NMuMG cells.

### Sample preparation for immunoprecipitation

For comparative interactome analyses, NMuMG cell clones were employed, including control wild-type clones, wild-type clones undergoing EMT, ZIP6 knockout clones undergoing EMT, and siRNA knockdowns of NCAM1 undergoing EMT. For each experiment, three biological replicates of each condition were maintained in parallel. For cells undergoing EMT, TGFB1 (240-B; R&D Systems, MN, USA) was added for 48 hrs at a concentration of 6.4 ng/mL and replenished with fresh medium after 24 hours. After 48 hrs of control or treatment conditions, medium was removed, cells were washed with ice cold PBS, and crosslinking was completed with a 15 min incubation with 2% formaldehyde in PBS. The formaldehyde was removed, and the reaction was quenched with a 10 min incubation with 125 mM glycine in PBS. Cells were again washed with ice cold PBS before undergoing lysis in an ice cold buffer consisting of 5 mM EGTA, 10% glycerol, 1% sodium deoxycholate, 1% NP-40, 150 mM HEPES (pH 8.0) and 1× Complete Protease Inhibitor Cocktail (11836170001; Roche). Insoluble cellular debris was cleared by centrifugation for 30 min at 4000 RPM and 4 °C.

For mapping the ZIP6-ZIP10 binding site of GSK3, cells were treated with TGFB1, 24 hours later transfected with plasmids coding for wild-type or internally deleted ZIP6 and/or ZIP10 expression plasmids, and 48 hours later *in vivo* crosslinked by 15 min exposure to 2% formaldehyde in PBS. The latter step was applied to stabilize protein-protein interactions prior to the disruption of cellular integrity. Cells lysis followed steps outlined above.

### Protein immunoprecipitation

Immunoprecipitation with ZIP6 and NCAM1 specific antibodies were completed with Protein A agarose (P3476; Sigma-Aldrich) and Protein G sepharose (17-0618-01; GE Healthcare) beads, respectively. Beads were transferred to a microcentrifuge tube and washed twice with ultrapure water and twice with PBS before the addition of the respective antibody, which had been diluted in PBS to fill the tube. Note that the large-scale and smaller-scale NCAM1 immunoprecipitations for analyses by mass spectrometry and *in vitro* GSK3B phosphorylation were undertaken with different antibodies, namely, the NCAM1-directed antibodies 556324 and OB11, respectively. The bead/antibody mixture was then gently agitated on a turning wheel for 4 hrs at room temperature (RT), before being equally divided into fresh tubes for individual samples. Beads were allowed to settle and the excess liquid was removed before protein samples were added. The samples were gently agitated on a turning wheel overnight at 4 °C, then washed thrice with 5 mM EGTA, 10% glycerol, 1% sodium deoxycholate, 1% NP-40, 150 mM HEPES (pH 8.0). Detergents were removed with two consecutive washes of 10 mM HEPES (pH 8.0), before samples were transferred to lo-bind 0.5 mL microcentrifuge tubes. Proteins were then eluted by acidification with 0.2% trifluoroacetic acid, 20% acetonitrile (pH 2.0).

The mapping of the GSK3 binding site to internal loop domains made use of affinity matrices based on the monoclonal capture antibody HA.11 (901501; BioLegend, CA, USA) bound to Protein G-sepharose (17-0618-01; GE Healthcare). The cell lysates were initially incubated with the antibody overnight and then added to beads for an overnight capture with gentle agitation at 4 °C. The proteins were at the end eluted with sample buffer and heating at 45 °C for 10 minutes.

### Active kinase assay

NCAM1 protein was immunoprecipitated with the OB11 antibody as described above and was *in vitro* phosphorylated with recombinant GSK3B while still attached to Protein G beads. More specifically, 4 μL (9 ng) of active GSK3B (catalog number 14-306, lot number WAA0024-B, Millipore, Burlington, Canada), 10 μL of hot ATP buffer, composed of 1 volume of [γ-^32^P]ATP (0.01 mCi) (catalog number NEG 502Z, Perkin-Elmer Inc., Woodbridge, ON, Canada) and 9 volumes of 75 mM MgCl_2_ and 500 μM ATP (catalog number A6559, Sigma-Aldrich, Oakville, ON, Canada), were added to 15 μL of NCAM1 immunoprecipitate to give rise to a reaction mix of 40 μL that was buffered by 8 mM MOPS, pH 7.0 and supplemented with 0.2 mM EDTA. After a 10 min incubation at 30 °C the reaction was stopped by the addition of Laemmli sample buffer. The analysis was undertaken in triplicate for wild-type and ZIP6 knockout cell extracts. As negative controls served samples that differed by the omission of GSK3B. Next, Protein G beads were pelleted by centrifugation, the supernatant containing the eluted proteins boiled for 15 min and loaded onto a large format isocratic (7.5%) SDS-PAGE gel that was cast in a Hoefer gel cassette. Incorporated radioactivity was revealed by overnight exposure of the SDS-PAGE gel to a double-emulsion X-ray film (catalog number Z363006, Carestream Kodak BioMax MS film, Sigma-Aldrich).

### Protein reduction, alkylation, trypsinization, and labeling

Eluted proteins were dried to a volume of 5 μL, washed with ultrapure water, and dried once again. Samples were then denatured by the addition of 9 M urea for 30 min at room temperature, before having disulfide bonds reduced for 30 min at 60 °C in the presence of 5 mM tris(2-carboxyethyl) phosphine (TCEP), and being alkylated for 1 hr at RT in the presence of 10 mM 4-vinylpyiridine (4-VP). Samples were then diluted with ultrapure water to ensure that the residual urea concentration of each sample was below 1.5 M to allow for digestion with side chain-modified porcine trypsin overnight at 37 °C. The covalent modification of peptides with reagents from the 6-plex amine-reactive tandem mass tag (TMT) (Thermo Fisher Scientific), or 8-plex isobaric tags for relative and absolute quantitation (iTRAQ) (4390733; AB Sciex, Concord, ON, Canada) labeling kits followed instructions provided by the manufacturers.

### Nanoscale HPLC-ESI tandem mass spectrometry

Immunoprecipitates were purified by C18 reversed-phase and strong cation exchange cartridges (Agilent Technologies, ON, Canada). Eluates were dried in a centrifugal concentrator then suspended in aqueous 0.1% formic acid and applied to C18 nanocapillary columns (25 cm long Acclaim PepMap RSLC with 100 Å pore size, 2 μm particle size, 75 μm inner diameter) using an EASY-nLC 1100 system (Thermo Fisher Scientific). Peptide separation was performed at 300 nl/minute using a binary mobile phase gradient with mobile phases containing 0.1% (v/v) formic acid in water or acetonitrile. Over the gradient, the organic content of the mobile phase was increased from 0 to 30% over 180 minutes then to 100% by 240 minutes. The nano-HPLC system was coupled by online nanoscale electrospray ionization (ESI) to an Orbitrap Fusion Tribrid mass spectrometer. The MS data acquisition method included three scan types. Each data acquisition cycle began with a survey scan of the 400–2000 m/z range in the Orbitrap at 120,000 resolution with automatic gain control set to 2e5 counts. Next, the most intense precursor ions carrying two to seven charges were separately isolated, subjected to collision-induced dissociation (CID) and their fragments detected in the linear ion trap. Finally MS3 with higher-energy collision-induced dissociation (HCD) was performed on the ten most intense fragments from each MS2 scan. The Orbitrap was used to obtain MS3 scans at 60,000 resolution with an automatic gain control target of 1e5 counts at a maximum injection time of 120 ms. The combined cycle time for this method was 3 seconds during which as many precursors as possible were analyzed. Dynamic exclusion prevented re-analysis of any precursor ion within a 20 ppm m/z window for 300 seconds.

### Protein identification and quantification

Peptide sequencing was performed using Mascot (Version 2.4; Matrix Science Ltd, London, UK) and Sequest HT search engines embedded in Proteome Discoverer software (Version 1.4; Thermo Fisher Scientific). The international protein index (IPI) mouse database (Version 3.87) was used as the protein sequence source in peptide-spectral matching. Only tryptic peptides with two or fewer missed cleavages were considered for assignment. Isobaric tags at peptide amino-termini and lysine residues were specified as fixed modifications. Variable modifications specified were methionine oxidation, asparagine and glutamine deamidation, cysteine pyridylethylation as well as phosphorylation at serine, threonine and tyrosine. For the Sequest HT searches, the number of identical modifications and dynamic modifications were limited to three and four per peptide respectively. The reliability of peptide-spectrum matches was assessed using the q-value determined by the Percolator algorithm at a false discovery rate (FDR) setting of 0.05. Peptide quantification was performed using MS3 data, in which reporter ion peaks were prevalent. The mass spectrometry proteomics data have been deposited to the ProteomeXchange Consortium via the PRIDE^41^ partner repository with the dataset identifier PXD004685.

### Statistical analyses

Error bars in data graphs represent the standard errors of the mean. For comparisons between two groups, statistical analyses were based on the paired Student’s *t*-test. A *p-*value of less than 0.05 was considered significant.

## Additional Information

**How to cite this article**: Brethour, D. *et al*. A ZIP6-ZIP10 heteromer controls NCAM1 phosphorylation and integration into focal adhesion complexes during epithelial-to-mesenchymal transition. *Sci. Rep.* **7**, 40313; doi: 10.1038/srep40313 (2017).

**Publisher's note:** Springer Nature remains neutral with regard to jurisdictional claims in published maps and institutional affiliations.

## Supplementary Material

Supplementary Information
