# Supplementary material for: A ZIP6-ZIP10 heteromer controls NCAM1 phosphorylation and integration into focal adhesion complexes during epithelial-to-mesenchymal transition
Source: Sci Rep. 2017 Jan 18;7:40313. doi: 10.1038/srep40313 (PMC5241876; doi:10.1038/srep40313)
Supplement: Supplementary Information [file srep40313-s1.pdf]

November 24, 2016

SUPPLEMENTARY INFORMATION

to manuscript titled

'A ZIP6-ZIP10 heteromer controls GSK3-dependent NCAM1 phosphorylation and integration into focal adhesion complexes during epithelial-to-mesenchymal transition'

Dylan Brethour[1,2], Mohadeseh Mehrabian[1,2], Declan Williams[1], Xinzhu Wang[1,2], Farinaz Ghodrati[1,2], Sepehr Ehsani[1,3], Elizabeth A. Rubie[4], James R. Woodgett[4], Jean Sevalle[1], Zhengrui Xi[1], Ekaterina Rogaeva[1,5], Gerold Schmitt-Ulms[1,2]*

[1]Tanz Centre for Research in Neurodegenerative Diseases, University of Toronto,
[2]Department of Laboratory Medicine & Pathobiology, University of Toronto, Ontario, Canada.
[3]Whitehead Institute for Biomedical Research, Cambridge, MA, USA.
[4]Lunenfeld-Tanenbaum Research Institute, Mount Sinai Hospital, Toronto, Ontario , Canada
[5]Department of Neurology, University of Toronto, Ontario, Canada.

Fig S1. Design and genomic validation of CRISPR-Cas9 nickase edited mouse ZIP6 knockout NMuMG cells.

(**a**) Exon and intron structure of mouse *Slc39a6.* (**b**) Targeting the mouse *Slc39a6* Exon 1 locus with a paired CRISPR-Cas9 nickase design using gRNAs ‘a’ and ‘b’. (**c**) Genomic sequence chromatograms of wt and ZIP6 ko clones with nucleotide residues missing in the ZIP6 ko clone highlighted in yellow. (**d**) Analysis of genomic PCR sequencing results with revealed that the 2-nucleotide deletion led to the formation of premature STOP codon. Note that due to the presence of additional introns downstream of the translation termination signal , the host-encoded nonsense-mediated decay program is expected to degrade the mutant transcripts, thereby largely precluding the formation of the truncated ZIP6 protein in the ZIP6 ko NMuMG clone.

Figure S1

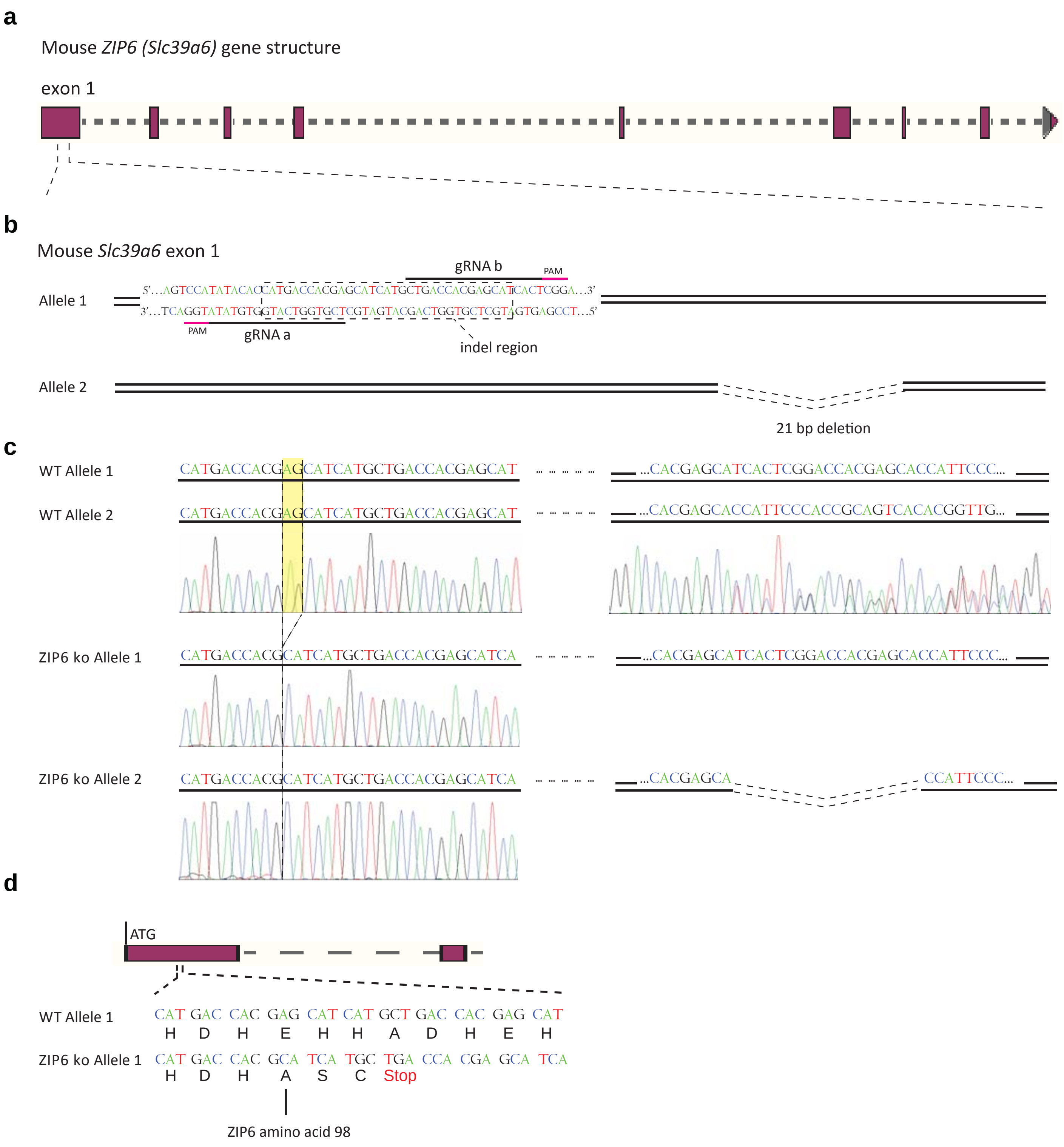
a
Mouse ZIP6 (Slc39a6) gene structure
exon 1
b
Mouse Slc39a6 exon 1
gRNA b
PAM
Allele 1
5'…AGTCCATATACACCATGACCACGAGCATCATGCTGACCACGAGCATCACTCGGA…3'
3'…TCAGGTATATGTGGTACTGGTGCTCGTAGTACGACTGGTGCTCGTAGTGAGCCT…5'
PAM
gRNA a
indel region
Allele 2
21 bp deletion
c
WT Allele 1
CATGACCACGAGCATCATGCTGACCACGAGCAT
…CACGAGCATCACTCGGACCACGAGCACCATTCCC…
WT Allele 2
CATGACCACGAGCATCATGCTGACCACGAGCAT
…CACGAGCACCATTCCCACCGCAGTCACACGGTTG…
ZIP6 ko Allele 1
CATGACCACGCATCATGCTGACCACGAGCATCA
…CACGAGCATCACTCGGACCACGAGCACCATTCCC…
ZIP6 ko Allele 2
CATGACCACGCATCATGCTGACCACGAGCATCA
…CACGAGCA
CCATTCCC…
d
ATG
WT Allele 1
CAT GAC CAC GAG CAT CAT GCT GAC CAC GAG CAT
H D H E H H A D H E H
ZIP6 ko Allele 1
CAT GAC CAC GCA TCA TGC TGA CCA CGA GCA TCA
H D H A S C Stop
ZIP6 amino acid 98

Table S1: The ZIP6 interactome in mouse NMuMG cells exhibiting a fibroblastoid morphology following 48 hrs of TGFB1 treatment

Accession	Description	Description (curated)	Coverage	Proteins	Unique	Total	PSMs	126/131	Var. [%]	127/131	Var. [%]	128/131	Var. [%]	129/131	Var. [%]	130/131	Var. [%]	Count	# AAs	MW [kDa]	calc. pI
IPI00469000.4	Zinc transporter ZIP6	Zinc transporter ZIP6	29.28%	2	10	23	101	9.085	427.2	1.000	0.0	7.790	654.8	1.000	0.0	7.976	324.9	27	765	86.3	6.84
IPI00989517.1	Uncharacterized protein (Fragment)	Neural Cell Adhesion Molecule 1	42.19%	7	6	21	59	13.952	94.7	1.000	58.1	13.132	75.2	1.000	47.2	10.828	45.8	11	839	92.3	4.88
IPI00123639.1	Calreticulin	Calreticulin	10.82%	1	1	6	19	8.139	6.0	0.393	16.7	10.486	11.3	0.863	1.5	7.846	7.6	3	416	48.0	4.49
IPI00273801.3	Zinc transporter ZIP10	Zinc transporter ZIP10	18.97%	2	11	16	78	6.653	113.4	1.000	7.0	7.012	219.1	1.000	0.0	5.972	104.3	31	833	94.3	6.71
IPI00111218.1	Aldehyde dehydrogenase, mitochondrial	Aldehyde dehydrogenase	42.00%	2	3	15	50	2.452	62.0	1.091	13.0	2.718	206.5	1.023	11.5	2.788	176.4	7	519	56.5	7.62
IPI00230108.6	Protein disulfide-isomerase A3	Protein disulfide-isomerase A3	30.30%	2	5	12	37	2.579	83.9	1.000	5.5	3.195	138.5	1.000	19.0	2.096	71.5	13	505	56.6	6.21
IPI00463589.2	Uncharacterized protein	ATPase, Ca++ transporting, plasma membrane 4	41.64%	5	6	33	56	3.026	128.1	1.000	0.0	1.971	74.9	1.000	1.8	1.612	19.5	7	1172	129.2	6.73
IPI00319994.6	L-lactate dehydrogenase A chain	L-lactate dehydrogenase A chain	44.88%	4	3	12	41	1.951	32.0	1.340	45.5	1.674	89.1	1.261	35.4	2.284	38.5	9	332	36.5	7.74
IPI00127841.3	ADP/ATP translocase 2	ADP/ATP translocase 2	42.62%	2	4	9	23	1.578	27.9	0.887	9.6	2.000	40.0	0.985	15.0	1.639	15.0	6	298	32.9	9.73
IPI00223060.1	Phosphoenolpyruvate carboxykinase [GTP], mitochondrial	Phosphoenolpyruvate carboxykinase	18.44%	2	5	10	24	1.475	42.4	1.146	46.9	1.613	72.0	1.134	59.1	1.786	88.6	13	667	73.4	7.74
IPI00135130.3	Neutral amino acid transporter A	Neutral amino acid transporter A	40.79%	1	9	16	156	1.541	26.7	0.950	32.2	1.858	37.5	0.896	16.4	1.458	29.4	49	532	56.0	5.87
IPI00624175.1	Isoform 1 of Lysophosphatidic acid phosphatase type 6	Lysophosphatidic acid phosphatase type 6	49.28%	4	8	17	58	1.794	38.1	0.991	34.6	1.559	38.0	0.960	26.3	1.496	32.2	20	418	47.6	7.72
IPI00122928.1	Tubulin beta-6 chain	Tubulin beta-6 chain	39.82%	2	2	13	38	1.481	20.9	0.882	43.4	1.596	55.3	0.921	27.9	1.575	59.6	6	447	50.1	4.89
IPI00554989.3	Peptidyl-prolyl cis-trans isomerase	Peptidyl-prolyl cis-trans isomerase	34.73%	5	3	7	9	1.795	30.6	0.920	16.2	1.551	17.9	0.783	25.2	1.246	31.4	3	167	18.3	7.90
IPI00622795.2	Glyceraldehyde-3-phosphate dehydrogenase	Glyceraldehyde-3-phosphate dehydrogenase	36.64%	28	3	13	52	1.389	45.8	0.926	11.4	1.748	20.3	0.889	103.2	1.418	39.2	13	333	35.8	8.03
IPI00117348.4	Tubulin alpha-1B chain	Tubulin alpha-1B chain	41.24%	4	1	14	53	1.555	19.0	1.057	13.7	1.520	20.1	1.003	21.9	1.424	30.3	20	451	50.1	5.06
IPI00110753.1	Tubulin alpha-1A chain	Tubulin alpha-1A chain	41.24%	5	1	13	53	1.474	19.7	1.048	14.9	1.490	20.2	1.003	21.9	1.378	31.7	20	451	50.1	5.06
IPI00117352.1	Tubulin beta-5 chain	Tubulin beta-5 chain	52.70%	6	5	21	57	1.234	32.3	0.977	34.5	1.587	68.6	0.885	38.5	1.493	48.7	13	444	49.6	4.89
IPI00874456.1	Dihydrolipoyl dehydrogenase, mitochondrial	Dihydrolipoyl dehydrogenase	36.94%	2	3	18	43	1.578	69.4	0.975	54.4	1.266	31.1	0.952	62.5	1.463	74.1	6	509	54.2	7.90
IPI00118108.1	Tetraspanin-6	Tetraspanin-6	21.22%	1	1	3	8	1.617	4.4	1.065	8.2	1.387	16.0	0.603	57.2	1.228	19.4	4	245	27.3	7.74
IPI00895479.1	Uncharacterized protein	KxDL motif-containing protein 1	53.02%	17	3	10	24	1.527	51.4	1.000	0.6	1.469	64.5	1.000	46.6	1.194	13.1	6	232	26.8	9.83
IPI00230351.1	Succinate dehydrogenase [ubiquinone] flavoprotein subunit, mitochondrial	Succinate dehydrogenase flavoprotein subunit	44.28%	1	4	24	64	1.399	62.2	1.083	36.8	1.402	48.4	0.949	70.2	1.257	19.7	9	664	72.5	7.37
IPI00229517.5	Galectin-1	Galectin-1	53.33%	1	2	6	15	1.658	29.2	1.035	43.1	1.318	30.7	0.980	33.9	1.080	18.2	4	135	14.9	5.49
IPI00407130.4	Isoform M2 of Pyruvate kinase isozymes M1/M2	Pyruvate kinase isozymes M1/M2	33.71%	4	5	17	41	1.262	20.0	0.955	61.3	1.362	44.6	1.243	33.5	1.370	31.2	10	531	57.8	7.47
IPI00121440.4	Electron transfer flavoprotein subunit beta	Electron transfer flavoprotein subunit beta	37.65%	1	3	9	18	1.173	23.1	0.750	18.2	1.427	1.8	0.949	16.1	1.390	18.6	4	255	27.6	8.10
IPI00399958.3	Calumenin isoform 2	Calumenin isoform 2	24.44%	6	3	6	20	1.493	38.5	1.095	15.9	1.208	16.2	0.970	145.1	1.283	56.1	7	315	37.1	4.59
IPI00331556.5	Heat shock 70 kDa protein 4	Heat shock 70 kDa protein 4	51.61%	1	10	36	104	1.453	30.5	0.972	14.8	1.279	22.4	0.964	22.3	1.224	30.7	23	841	94.1	5.24
IPI00338536.1	Succinate dehydrogenase [ubiquinone] iron-sulfur subunit, mitochondrial	Succinate dehydrogenase iron-sulfur subunit	60.99%	2	5	20	53	1.287	12.1	1.090	20.8	1.361	26.8	1.089	23.6	1.298	13.9	7	282	31.8	8.68
IPI00263863.8	10 kDa heat shock protein, mitochondrial	10 kDa heat shock protein	80.39%	3	6	10	46	1.403	37.2	0.859	22.0	1.233	22.5	0.846	26.9	1.305	16.8	17	102	11.0	8.35
IPI00323592.2	Malate dehydrogenase, mitochondrial	Malate dehydrogenase	84.32%	1	11	23	66	1.252	22.8	0.961	13.9	1.322	23.0	0.917	14.3	1.338	16.5	20	338	35.6	8.68
IPI00875833.2	Monofunctional C1-tetrahydrofolate synthase, mitochondrial	Monofunctional C1-tetrahydrofolate synthase	43.50%	1	11	38	90	1.064	37.7	1.000	31.5	1.251	35.7	0.903	28.2	1.492	25.3	18	977	105.7	7.02
IPI00117083.1	GrpE protein homolog 1, mitochondrial	GrpE protein homolog 1	26.73%	1	6	7	12	1.190	29.3	0.951	33.6	1.318	91.0	0.951	61.0	1.231	21.5	7	217	24.3	8.38
IPI00454049.4	Enoyl-CoA hydratase, mitochondrial	Enoyl-CoA hydratase	58.97%	3	13	17	146	1.082	23.1	0.850	22.1	1.384	15.2	0.944	21.6	1.220	22.2	47	290	31.5	8.48
IPI00648141.1	Glycogen synthase kinase-3 alpha	Glycogen synthase kinase-3 alpha	50.61%	13	6	19	88	1.056	16.6	1.000	31.3	1.404	28.3	0.979	10.6	1.177	24.5	24	490	51.6	8.81
IPI00318614.9	Isocitrate dehydrogenase [NADP], mitochondrial	Isocitrate dehydrogenase [NADP]	31.19%	2	7	13	35	1.156	21.7	0.923	19.4	1.225	14.7	1.000	31.7	1.245	26.8	11	452	50.9	8.69
IPI00114209.1	Glutamate dehydrogenase 1, mitochondrial	Glutamate dehydrogenase 1	35.66%	2	11	20	43	1.092	16.6	0.937	9.6	1.125	17.9	0.965	36.6	1.383	35.8	14	558	61.3	8.00
IPI00671957.2	Glutaminase isoform 2	Glutaminase isoform 2	52.90%	5	6	18	49	1.196	38.0	1.020	40.6	1.157	50.8	0.884	25.2	1.237	48.1	14	603	66.0	8.12
IPI00125319.1	Glycogen synthase kinase-3 beta	Glycogen synthase kinase-3 beta	50.48%	5	6	17	116	1.235	33.5	0.852	29.3	1.220	30.2	1.000	29.3	1.111	24.1	36	420	46.7	8.78
IPI00308885.6	Isoform 1 of 60 kDa heat shock protein, mitochondrial	60 kDa heat shock protein	64.75%	4	19	35	193	1.127	16.5	0.950	14.6	1.201	19.9	0.821	23.6	1.219	16.5	60	573	60.9	6.18
IPI00111960.2	Lysosomal alpha-glucosidase	Lysosomal alpha-glucosidase	28.23%	5	5	19	46	1.270	7.7	1.079	21.8	1.170	12.5	0.937	12.6	1.103	17.2	9	953	106.2	5.83
IPI00113141.1	Citrate synthase, mitochondrial	Citrate synthase	38.36%	1	4	19	52	1.111	23.3	0.902	24.0	1.202	11.7	0.865	15.2	1.229	16.9	12	464	51.7	8.57
IPI00116074.1	Aconitate hydratase, mitochondrial	Aconitate hydratase	39.10%	1	13	25	82	1.133	32.3	0.832	26.8	1.216	24.5	0.930	25.5	1.164	26.2	24	780	85.4	7.93
IPI00118825.2	Citrate synthase-like protein	Citrate synthase-like protein	43.13%	1	3	18	57	1.174	34.7	1.002	21.4	1.151	20.2	0.916	18.6	1.173	19.2	8	466	52.3	8.79
IPI00319992.1	78 kDa glucose-regulated protein	78 kDa glucose-regulated protein	30.38%	1	10	21	109	1.045	27.0	1.000	25.9	1.229	35.3	1.034	29.6	1.167	27.5	30	655	72.4	5.16
IPI00117312.1	Aspartate aminotransferase, mitochondrial	Aspartate aminotransferase	43.02%	1	7	14	73	1.160	24.9	1.000	20.5	1.061	23.8	0.895	17.2	1.194	18.1	27	430	47.4	9.00
IPI00459725.2	Isoform 1 of Isocitrate dehydrogenase [NAD] subunit alpha, mitochondrial	Isocitrate dehydrogenase [NAD] subunit alpha	32.51%	2	6	12	36	1.130	45.2	0.873	17.7	1.130	39.5	0.896	15.7	1.116	9.4	10	366	39.6	6.73

Table S2: The NCAM interactome in mouse NMuMG cells exhibiting a fibroblastoid morphology following 48 hrs of TGFB1 treatment

Accession	Description	Description (curated)	Coverage	Proteins	Unique	Total	PSMs	113/121	Var. [%]	115/121	Var. [%]	117/121	Var. [%]	119/121	Var. [%]	Count	# AAs	MW [kDa]	calc. pI
IPI00230665.3	Neural cell adhesion molecule 1 isoform 3	Neural cell adhesion molecule 1 isoform 3	67.62%	5	10	70	1785	8.763	377.4	8.676	467.0	11.261	315.4	1.000	0.0	518	1115	119.2	4.82
IPI00989517.1	Uncharacterized protein (Fragment)	Neural cell adhesion molecule 1	68.65%	5	2	60	1414	9.891	395.3	9.912	471.0	12.823	261.6	1.000	0.0	414	839	92.3	4.88
IPI00408904.3	NCAM	NCAM	79.59%	1	1	4	37	7.542	261.9	4.974	1688.9	14.365	79.0	1.000	31.6	16	49	5.2	8.68
IPI00650039.1	Profilin	Profilin	52.68%	2	3	4	12	17.584	128.2	28.256	43.4	31.035	42.9	1.000	45.3	5	112	11.8	4.88
IPI00319994.6	L-lactate dehydrogenase A chain	L-lactate dehydrogenase A chain	39.76%	5	6	16	45	16.573	60.8	24.680	80.8	20.156	70.4	1.000	0.0	9	332	36.5	7.74
IPI00120045.1	MCG8024	Heat Shock 10kDa Protein 1	48.04%	2	3	5	14	9.880	124.3	13.300	55.9	11.298	64.7	1.000	21.6	6	102	11.0	8.92
IPI00330862.5	Ezrin	Ezrin	20.99%	4	1	12	22	11.141	40.6	10.222	54.9	11.993	58.2	1.150	32.1	6	586	69.4	6.10
IPI00132474.3	Integrin beta-1	Integrin beta-1	23.31%	1	6	13	50	12.877	584.9	10.429	229.4	9.324	266.7	1.000	0.0	18	798	88.2	5.94
IPI00987580.1	Peptidyl-prolyl cis-trans isomerase	Peptidyl-prolyl cis-trans isomerase	57.93%	2	1	8	27	9.962	566.6	11.532	556.0	8.888	492.3	1.259	36.9	8	164	18.0	6.68
IPI01008196.1	NADH dehydrogenase [ubiquinone] iron-sulfur protein 4, mitochondrial	NADH dehydrogenase iron-sulfur protein 4	36.57%	2	1	6	14	11.016	144.9	9.821	49.2	9.461	160.2	1.000	0.0	3	175	19.8	9.99
IPI00126077.2	Integrin alpha-2	Integrin alpha-2	25.47%	1	8	22	103	8.817	182.7	8.824	295.4	12.239	149.3	1.000	0.0	28	1178	128.9	5.21
IPI00229517.5	Galectin-1	Galectin-1	34.81%	1	3	4	36	9.183	89.6	9.761	141.9	10.845	166.0	1.001	0.8	10	135	14.9	5.49
IPI00457898.3	Phosphoglycerate mutase 1	Phosphoglycerate mutase 1	30.31%	2	3	7	14	8.610	47.1	9.910	54.8	10.935	41.5	1.587	77.4	6	254	28.8	7.18
IPI00121427.1	Protein S100-A6	Protein S100-A6	22.47%	1	2	3	9	8.172	18.7	10.540	6.5	10.295	8.7	1.476	16.1	6	89	10.0	5.48
IPI00116498.1	14-3-3 protein zeta/delta	14-3-3 protein zeta/delta	21.63%	10	2	5	10	7.345	144.7	10.139	13.3	9.462	93.8	1.000	28.2	3	245	27.8	4.79
IPI00554989.3	Peptidyl-prolyl cis-trans isomerase	Peptidyl-prolyl cis-trans isomerase	69.46%	2	1	10	35	7.171	114.5	9.133	78.5	8.888	60.3	1.615	81.0	10	167	18.3	7.90
IPI00462072.3	Alpha-enolase	Alpha-enolase	75.35%	15	14	24	186	6.778	230.9	9.169	167.6	9.054	243.0	1.000	6.2	65	434	47.1	6.80
IPI00853924.1	Uncharacterized protein	14-3-3 protein theta	34.98%	13	1	8	17	6.185	25.9	9.300	12.9	9.130	5.3	0.851	3.7	3	303	34.3	6.38
IPI00987093.1	Uncharacterized protein	ADP-Ribosylation Factor 4	51.38%	5	1	7	18	9.215	18.7	8.904	75.5	4.537	46.5	2.011	130.6	3	181	20.4	6.16
IPI00969323.2	Polyubiquitin-C	Polyubiquitin-C	66.62%	16	3	9	26	7.507	77.6	6.678	89.6	7.545	64.3	1.000	0.0	8	734	82.5	8.62
IPI00468203.3	Annexin A2	Annexin A2	46.61%	4	9	18	67	5.738	168.1	6.348	210.8	6.080	414.7	1.000	26.3	25	339	38.7	7.69
IPI00311682.5	Sodium/potassium-transporting ATPase subunit alpha-1	Sodium/potassium-transporting ATPase subunit alpha-1	23.46%	8	7	18	43	3.378	499.8	5.494	204.1	7.893	136.9	1.000	0.0	14	1023	112.9	5.45
IPI00115564.5	ADP/ATP translocase 1	ADP/ATP translocase 1	46.98%	2	1	14	41	5.423	219.9	5.611	121.8	5.597	160.3	1.000	10.4	10	298	32.9	9.72
IPI00123996.2	Neuropilin-1	Neuropilin-1	11.48%	1	4	9	34	4.175	150.1	3.859	124.6	5.249	86.1	1.000	0.0	8	923	102.9	5.90
IPI00130280.1	ATP synthase subunit alpha, mitochondrial	ATP synthase subunit alpha	44.30%	4	9	22	73	3.162	220.6	5.105	92.4	4.855	97.0	1.028	44.0	23	553	59.7	9.19
IPI00323592.2	Malate dehydrogenase, mitochondrial	Malate dehydrogenase	55.92%	1	4	15	33	4.041	200.9	4.883	138.9	3.616	179.4	1.118	16.6	8	338	35.6	8.68
IPI00127841.3	ADP/ATP translocase 2	ADP/ATP translocase 2	47.65%	2	3	15	61	4.353	156.3	3.916	173.5	4.159	199.2	1.000	0.0	19	298	32.9	9.73
IPI00848816.1	Cofilin-1-like	Cofilin-1-like	45.78%	4	5	7	31	3.613	147.4	4.171	184.3	3.696	198.7	1.000	0.0	14	166	18.5	7.74
IPI00856379.1	Fructose-bisphosphate aldolase A isoform 1	Fructose-bisphosphate aldolase A isoform 1	57.42%	8	8	17	87	3.141	338.0	3.439	456.5	4.127	273.4	1.000	0.0	27	418	45.1	7.91
IPI00133522.2	Protein disulfide-isomerase	Protein disulfide-isomerase	29.67%	1	5	12	35	3.964	417.0	3.040	376.1	2.960	350.9	1.000	0.0	8	509	57.0	4.88
IPI00622235.5	Transitional endoplasmic reticulum ATPase	Transitional endoplasmic reticulum ATPase	29.65%	1	5	14	38	2.719	283.1	3.638	281.3	3.534	228.4	1.000	0.0	9	806	89.3	5.26
IPI00307837.6	Elongation factor 1-alpha 1	Elongation factor 1-alpha 1	39.18%	4	8	12	35	2.993	112.3	2.597	141.1	4.070	156.2	0.991	5.7	16	462	50.1	9.01
IPI00986866.1	Uncharacterized protein	CD166 antigen	26.32%	4	2	9	22	2.450	220.0	2.259	181.8	4.516	159.8	1.000	0.0	6	570	63.6	7.27
IPI00849045.1	Glyceraldehyde-3-phosphate dehydrogenase	Glyceraldehyde-3-phosphate dehydrogenase	42.94%	27	5	9	46	2.894	98.0	2.455	82.9	3.601	101.7	1.000	9.3	13	333	35.8	8.44
IPI00930882.1	4F2 cell-surface antigen heavy chain isoform a	4F2 cell-surface antigen heavy chain	24.07%	2	3	8	18	3.513	506.8	1.714	94.5	3.574	452.9	1.000	0.0	6	565	62.2	5.50
IPI00652436.3	Putative uncharacterized protein	Actin Gamma 1	55.47%	16	4	17	141	2.714	281.9	3.078	388.4	2.592	251.9	1.000	2.4	54	375	41.7	5.38
IPI00554929.3	Heat shock protein HSP 90-beta	Heat shock protein HSP 90-beta	25.28%	2	3	14	28	2.037	142.9	2.464	223.0	3.498	163.9	1.000	0.0	7	724	83.2	5.03
IPI00230395.5	Annexin A1	Annexin A1	36.42%	2	4	11	32	3.336	482.8	2.242	178.6	2.020	140.1	1.000	39.3	9	346	38.7	7.37
IPI00110827.1	Actin, alpha skeletal muscle	Actin, alpha skeletal muscle	61.80%	11	1	17	91	2.046	144.4	2.654	266.9	2.685	274.6	1.201	27.7	29	377	42.0	5.39
IPI00110588.4	Moesin	Moesin	51.82%	4	8	26	94	2.663	221.3	2.147	161.6	2.210	172.7	1.000	0.0	25	577	67.7	6.60
IPI00752080.1	Uncharacterized protein	Integrin, alpha V	43.15%	3	14	34	113	2.958	350.3	2.019	140.1	2.035	142.6	1.000	0.0	38	1008	111.4	5.67
IPI00308885.6	Isoform 1 of 60 kDa heat shock protein, mitochondrial	60 kDa heat shock protein	23.21%	2	8	9	52	2.352	199.7	1.000	0.0	3.326	478.6	1.000	0.0	17	573	60.9	6.18
IPI00845840.1	Isoform M1 of Pyruvate kinase isozymes M1/M2	Pyruvate kinase isozymes M1/M2	43.69%	4	7	16	45	2.366	202.6	1.473	62.5	2.823	220.4	1.000	0.0	15	531	57.9	7.14
IPI00331182.4	Uncharacterized protein	Glycerol-3-phosphate dehydrogenase 2	45.77%	1	17	24	108	2.793	302.8	1.675	89.2	2.114	155.8	1.000	0.0	41	745	82.8	6.42
IPI00132575.3	Coactosin-like protein	Coactosin-like protein	42.25%	1	3	5	43	1.000	0.2	1.433	90.0	3.710	48.0	1.000	43.1	11	142	15.9	5.40
IPI00468481.2	ATP synthase subunit beta, mitochondrial	ATP synthase subunit beta	40.08%	1	9	15	62	2.224	175.2	1.618	81.5	2.277	185.1	1.000	5.4	22	529	56.3	5.34
IPI00122257.1	5'-nucleotidase	5'-nucleotidase	15.63%	2	4	6	35	2.106	154.5	1.732	97.0	2.263	182.5	1.000	0.0	10	576	63.8	6.64
IPI00608020.4	Ferritin light chain 1	Ferritin light chain 1	55.74%	4	2	8	25	1.682	90.2	1.404	53.7	2.783	255.6	1.000	0.0	8	183	20.8	6.00
IPI00653231.1	Sideroflexin-3 isoform 3	Sideroflexin-3 isoform 3	49.11%	3	3	8	14	1.408	12.8	2.635	37.5	1.814	55.3	1.000	0.0	5	281	30.9	8.98
IPI00221613.5	ADP-ribosylation factor 1	ADP-ribosylation factor 1	41.44%	3	1	5	22	1.582	40.1	1.631	83.2	2.047	67.2	1.000	0.0	4	181	20.7	6.80
IPI00323357.3	Heat shock cognate 71 kDa protein	Heat shock cognate 71 kDa protein	50.93%	6	11	26	210	1.150	70.8	1.614	81.0	2.344	96.0	1.043	59.0	48	646	70.8	5.52
IPI00988063.1	Triosephosphate isomerase	Triosephosphate isomerase	35.74%	2	1	5	15	1.465	61.4	1.711	94.1	1.703	47.6	1.000	0.0	4	249	26.7	7.30
IPI00124640.4	Granulins	Granulins	16.78%	1	1	8	19	0.897	46.9	1.763	53.5	1.493	61.0	0.714	20.3	8	602	65.0	6.99
IPI00309230.3	Beta-glucuronidase	Beta-glucuronidase	8.02%	4	1	4	10	1.482	19.9	1.047	6.8	1.531	70.1	1.283	38.3	4	648	74.2	6.64
IPI00110753.1	Tubulin alpha-1A chain	Tubulin alpha-1A chain	46.78%	8	4	12	62	1.453	60.0	1.000	33.2	1.462	61.1	1.000	42.3	27	451	50.1	5.06
IPI00319992.1	78 kDa glucose-regulated protein	78 kDa glucose-regulated protein	39.24%	1	16	22	142	1.357	47.7	1.000	0.9	1.469	62.0	1.000	0.0	53	655	72.4	5.16
IPI00470167.3	Reticulon-4 isoform B1	Reticulon-4 isoform B1	53.65%	5	8	10	50	1.511	67.3	1.000	0.0	1.272	36.8	1.000	0.0	25	356	38.4	4.78
